# Specific Phospholipid Modulation by Muscarinic Signaling in a Rat Lesion Model of Alzheimer’s Disease

**DOI:** 10.1021/acschemneuro.1c00169

**Published:** 2021-05-26

**Authors:** Alberto Llorente-Ovejero, Jonatan Martínez-Gardeazabal, Marta Moreno-Rodríguez, Laura Lombardero, Estíbaliz González de San Román, Iván Manuel, María Teresa Giralt, Rafael Rodríguez-Puertas

**Affiliations:** † Department of Pharmacology, Faculty of Medicine and Nursing, University of the Basque Country (UPV/EHU), B° Sarriena s/n, 48940 Leioa, Spain; ‡ Neurodegenerative Diseases, BioCruces Bizkaia Health Research Institute, 48903 Barakaldo, Spain

**Keywords:** Cholinergic, 192IgG-saporin, autoradiography, muscarinic receptor, lipid, MALDI

## Abstract

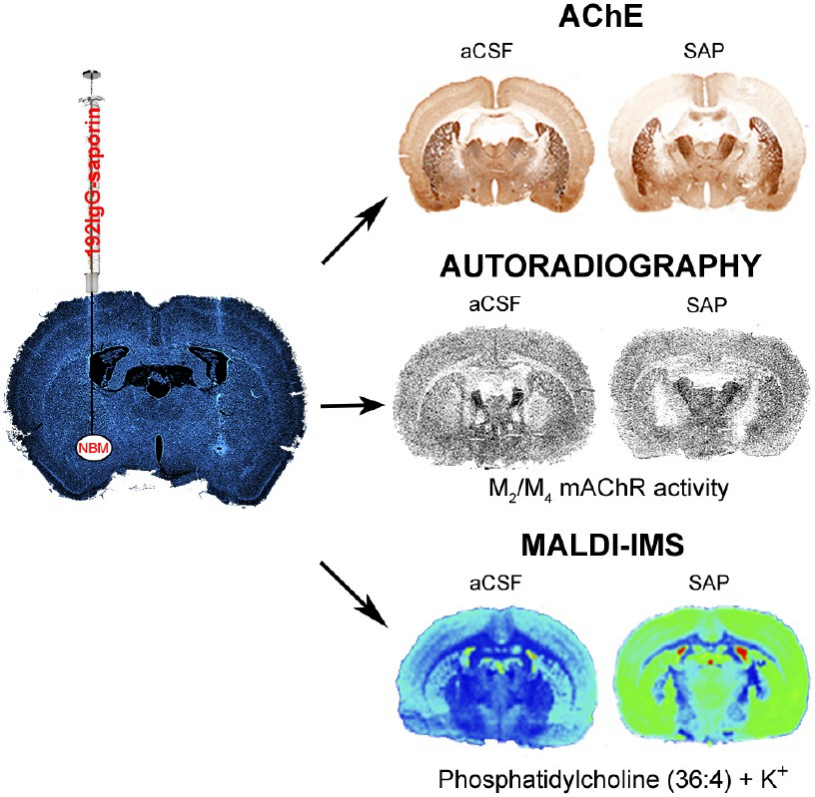

Alzheimer’s disease (AD) represents the most common cause of dementia worldwide and has been consistently associated with the loss of basal forebrain cholinergic neurons (BFCNs) leading to impaired cholinergic neurotransmission, aberrant synaptic function, and altered structural lipid metabolism. In this sense, membrane phospholipids (PLs) can be used for de novo synthesis of choline (Ch) for the further obtaining of acetylcholine (ACh) when its availability is compromised. Specific lipid species involved in the metabolism of Ch have been identified as possible biomarkers of phenoconversion to AD. Using a rat model of BFCN lesion, we have evaluated the lipid composition and muscarinic signaling in brain areas related to cognitive processes. The loss of BFCN resulted in alterations of varied lipid species related to Ch metabolism at nucleus basalis magnocellularis (NMB) and cortical projection areas. The activity of muscarinic receptors (mAChR) was decreased in the NMB and increased in the hippocampus according to the subcellular distribution of M_1_/M_2_ mAChR which could explain the learning and memory impairment reported in this AD rat model. These results suggest that the modulation of specific lipid metabolic routes could represent an alternative therapeutic strategy to potentiate cholinergic neurotransmission and preserve cell membrane integrity in AD.

## Introduction

Alzheimer’s disease (AD) is the most frequent irreversible form of dementia, characterized by a progressive cognitive impairment. The specific vulnerability of the cholinergic system at the basalocortical pathway has been extensively described in AD. This decline includes the loss of basal forebrain cholinergic neurons (BFCNs), decrease in choline acetyltransferase (ChAT) activity and density of the high-affinity choline uptake carrier, and altered density of muscarinic receptors (mAChR), leading to severe cholinergic neurotransmission deficiencies.^1−5^ Changes in cholinergic neurotransmission were identified as responsible for the short-term learning deficits and memory loss described at the onset of adult dementia disorders.^6^ Moreover, the activity of muscarinic antagonists inducing amnesia and cognitive impairment was also described decades ago.^7^ Altogether, they contributed to formulate the cholinergic hypothesis of geriatric memory dysfunction at that time.^8^ The biosynthesis of acetylcholine (ACh) critically depends on the levels of choline (Ch).^9^ Ch can be obtained mainly from the hydrolysis of ACh, presynaptic uptake, and circulating Ch in the bloodstream and derived from the hydrolysis of membrane phospholipids at the expense of membrane formation when other sources are compromised.^10^ This metabolic source may finally impair synaptic plasticity or compromise membrane viability leading to autophagy mechanisms as an adaptive response to stress provoked by the cholinergic dysfunction described in the neurodegenerative process of AD.^11−14^

The loss of BFCNs leads to a decrease in the production and release of ACh in both the basal cholinergic forebrain and in innervated cortical areas, with the consequent reduction in the pool of Ch available for ACh synthesis. The BFCNs from the nucleus basalis magnocellularis (NBM) innervate most of the cerebral cortex, provide the main source of cortical ACh, and control learning and memory processes. In this regard, 192IgG-saporin (SAP), an immunotoxin directed against the low affinity nerve growth factor receptor p75NTR, mainly expressed in BFCN, represents a powerful tool to specifically eliminate those cells to mimic the cholinergic degeneration described in AD.^15^ The intraparenchymal infusion of 192IgG-saporin in the NBM of rats decreases ChAT and acetylcholinesterase (AChE) levels, reduces the density of the high-affinity Ch transporter, and modifies the activity and cellular distribution of mAChR (see review from ref (16)). Consequently, the loss of BFCN impairs learning and memory.^17^ Intraparenchymal injection of SAP in the NBM results in deficits in recognition memory capacity, in delayed matching to position, association learning, and aversive learning and memory. Attentional functions are also impaired including vigilance, reorienting of spatial attention, and attentional resources directed at environmental stimuli.^18,19^ Previous results clearly demonstrated that the protocol used in the present study is able to induce deficits in aversive learning,^17^ spatial memory by using the Barnes maze as a nonaversive spatial navigation test,^20^ or learning and memory related to novelty-seeking by novel object recognition test (unpublished results).

Lipids (mainly phospholipids; PL), which are the main structural components of membranes, are also involved in the control of multiple signaling pathways and energy metabolism. Different studies have described changes in the abundance and distribution of several of these lipid species in AD.^21−27^ Several attempts to alleviate the deficit of ACh in AD patients by the administration of cholinergic enhancers or precursors such as Ch or lecithin have failed, while those based on phosphatidylserine, Ch-alphoscerate, and CDP-Ch displayed slight benefits (reviewed in ref (28)). Despite the limited therapeutic effects of cholinergic enhancers, the AChE inhibitors together with memantine continue being the only approved drugs available for AD treatment.^29^ These treatments are not able to reverse the progressive cholinergic neurodegeneration as the primary cause is not treated since it has not been identified. However, the direct modulation of phospholipids involved in neurotransmission signaling may represent an alternative therapeutic strategy to potentiate cholinergic neurotransmission in AD. Currently, it is possible to anatomically localize the diversity and specialization of lipid content in discrete brain areas of humans as well as in rodents by using matrix-assisted laser desorption ionization-imaging mass spectrometry (MALDI-IMS).^27,30^ The combination of this technique with neurochemical and behavioral studies is already contributing to the understanding of the physiological role of specific lipid species in the underlying pathological mechanisms involved in cognitive impairment.^31^

Thus, the present study analyzes by MALDI-IMS the modulation mediated by mAChR signaling of specific PL in the basalocortical pathway following the specific depletion of BFCN to identify a possible alternative restoration of cholinergic neurotransmission during AD progression.

## Results and Discussion

### Learning and Memory Impairment after Basal Forebrain Cholinergic Lesion

**Figure 1:**
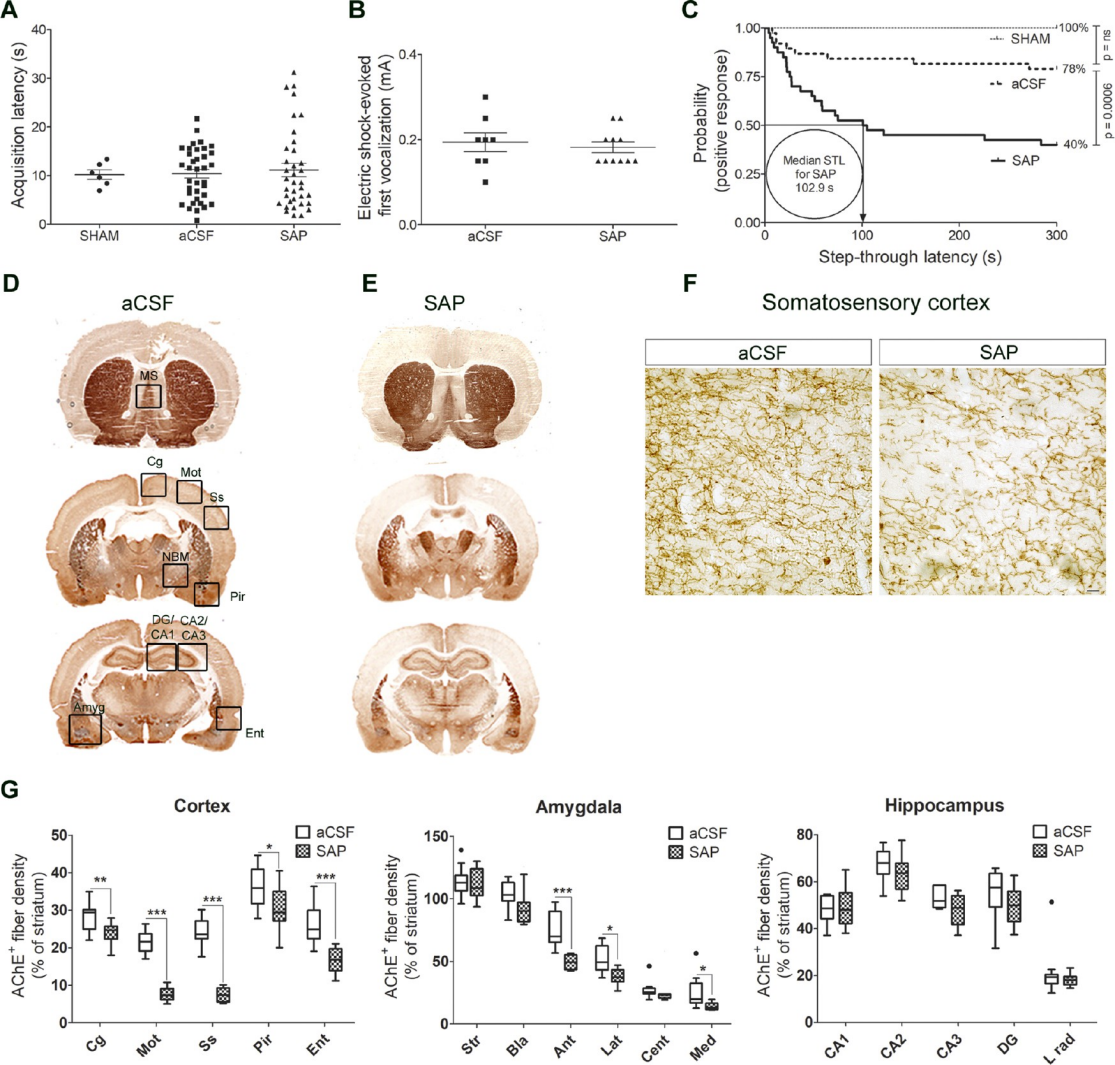
(A) Acquisition latency times in the learning trial of the passive avoidance test of SHAM (*n* = 6), aCSF (*n* = 36), and SAP (*n* = 36) rats. (B) Electrical intensity (mA) required to provoke the first audible vocalization when delivering increasing intensities of foot-shock to aCSF (*n* = 8) and SAP (*n* = 11) rats (*p* = ns aCSF vs SAP). (C) Step-through latency times in the retention trial, represented as Kaplan–Meier survival curves. The median step-through latency or the time spent to enter the dark compartment by 50% of the immunotoxin-treated rats was calculated in 102.9 s (aCSF vs SHAM, *p* = ns; SAP vs aCSF *p* < 0.001, log-rank test). (D, E) AChE staining in brain sections at three different levels of aCSF and SAP-treated rats. (F) Microphotographs from somatosensory cortex of aCSF and SAP-treated rats at 400-fold magnification, revealing decreased AChE positive fibers in the immunotoxin-treated rats (scale bar = 20 μm). (G) Optical density of AChE expressed as percentages of the striatal levels (used as control area) in aCSF (*n* = 13) and SAP (*n* = 13) treated rats: *p < 0.05, **p < 0.01, and ***p < 0.001 SAP vs aCSF-treated rats. Outliers are indicated by black dots or circles in the G panel. MS: medial septum. Cg: cingulate cortex. Mot: motor cortex. Ss: somatosensory cortex. Pir: piriform cortex. Ent: entorhinal cortex. Amyg: amygdaloid nuclei (Bla, basolateral; Ant, anterior; Lat, lateral; Cent, central; Med, medial). DG: dentate gyrus. CA1-CA2-CA3: hippocampal CA1-CA2-CA3 regions. Str: striatum.

One week after the administration of the immunotoxin 192IgG-saporin, cognitive functions related to learning and memory processes were evaluated in the passive avoidance test (PA) (Figure 1A). The results of a study carried out in a separate group of animals are shown to validate the usefulness of the PA test in this rat model of AD, since recent studies demonstrated that the modulation of cholinergic neurotransmission modifies the perception of pain when using nociceptive stimuli-based learning tests.^32^ Comparable electric-shock-evoked first vocalizations (pain threshold) between vehicle (artificial cerebrospinal fluid; aCSF) and lesioned (SAP) treated groups (*p* = ns vs aCSF) were observed when electric shocks of increasing intensity were delivered (Figure 1B), which demonstrates for the first time that cholinergic neurotransmission arising from NBM does not substantially contribute to bias the perception of pain in the PA test.

Then, the animals used for the neurochemical studies were tested in the PA. The phase of acquisition or learning revealed no differences between aCSF and SAP groups (Figure 1A). Twenty-four hours later, rats were again tested to evaluate memory and retrieval of the aversive stimulus by measuring the step-through latency. On the basis of previous reports, Kaplan–Meier survival curves were used to determine and graphically represent the probability of a positive response (i.e., reaching the cutoff time of 300 s).^17,33^ When the three groups were compared, 100% and 78% of control (SHAM) and aCSF-treated rats, respectively (p = ns), remembered the aversive stimulus, while only 40% of immunotoxin-treated rats displayed a positive response (*p* = 0.0001 vs SHAM and *p* = 0.0006 vs aCSF) which showed a median latency time of 102.9 s for SAP-treated rats (Figure 1C). The negative behavioral responses in the PA test following the depletion of approximately 80% of BFCN are consistent with the observations of other authors.^30,34,35^

AChE enzymatic assay was used to verify the status of the cholinergic lesion induced by the 192IgG-saporin. SHAM-operated rats showed equivalent levels of AChE staining to those measured in the aCSF-treated group (data not shown). SAP did not modify the AChE+ fiber density in the striatum but resulted in an extensive reduction in the entire cortical mantle, ranging from 20% in cingular (*p* < 0.01) and piriform (*p* < 0.05) to 60–70% in motor and entorhinal cortices (*p* < 0.001), indicating the specificity of the lesion to BFCN of the NBM. No differences were observed in the medial septum or hippocampus, revealing additional evidence of the absence of nonspecific damage in other basal forebrain cholinergic projection pathways (Figure 1D,G). A previous study showed that the loss of 50% of BFCN in the NBM leads to a 25–30% decrease in ChAT activity^36^ which is consistent with the present data.

### Modulation of the Lipid Profile in NBM and Cortex Induced by BFCN Lesion

The MALDI-IMS technique allowed us to anatomically localize and quantify several lipid species in tissue sections. In this study, a lipidomic analysis was carried out in coronal brain sections including the NBM and several cortical regions, from aCSF or SAP groups in both positive and negative ion detection modes. The PL composition of potential Ch precursors was specifically analyzed in the lesion site and cortical projections.

**Figure 2:**
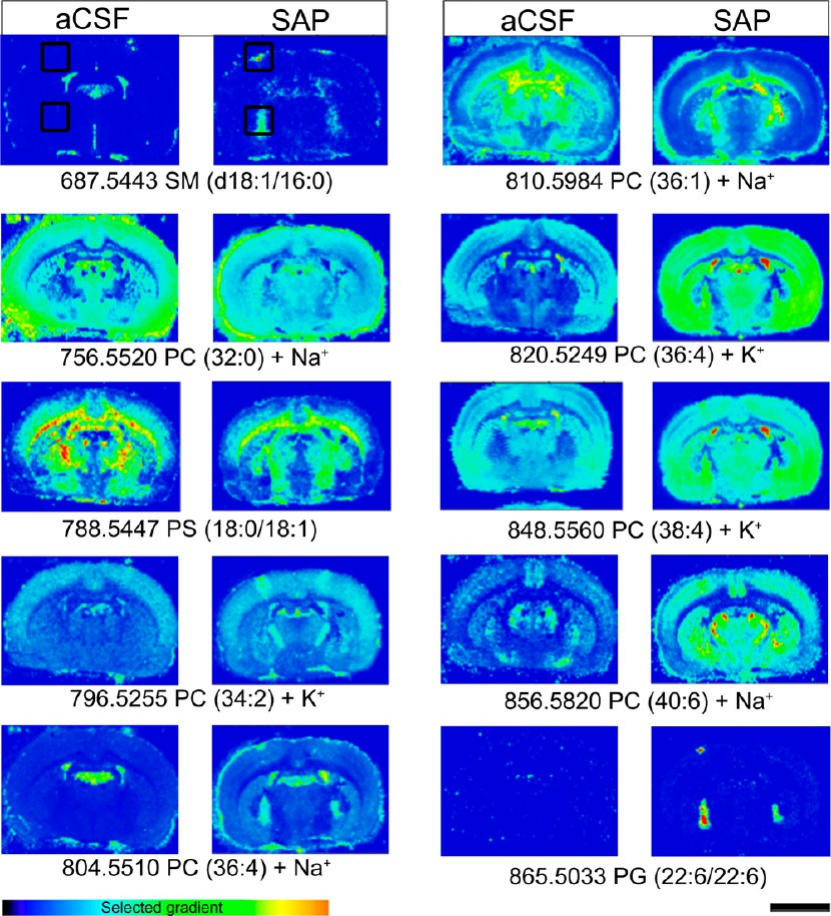
Matrix-assisted laser desorption ionization imaging mass spectrometry (MALDI-IMS) of different lipids in brain slices containing the NBM and cortical projections from aCSF (*n* = 8) and SAP-treated rats (*n* = 8) which show marked differences in the distribution of certain lipid species following the cholinergic lesion. The intensities were measured in the areas marked with black squares of the somatosensory cortex and the NBM. Scale bar = 5 mm.

**Figure 3:**
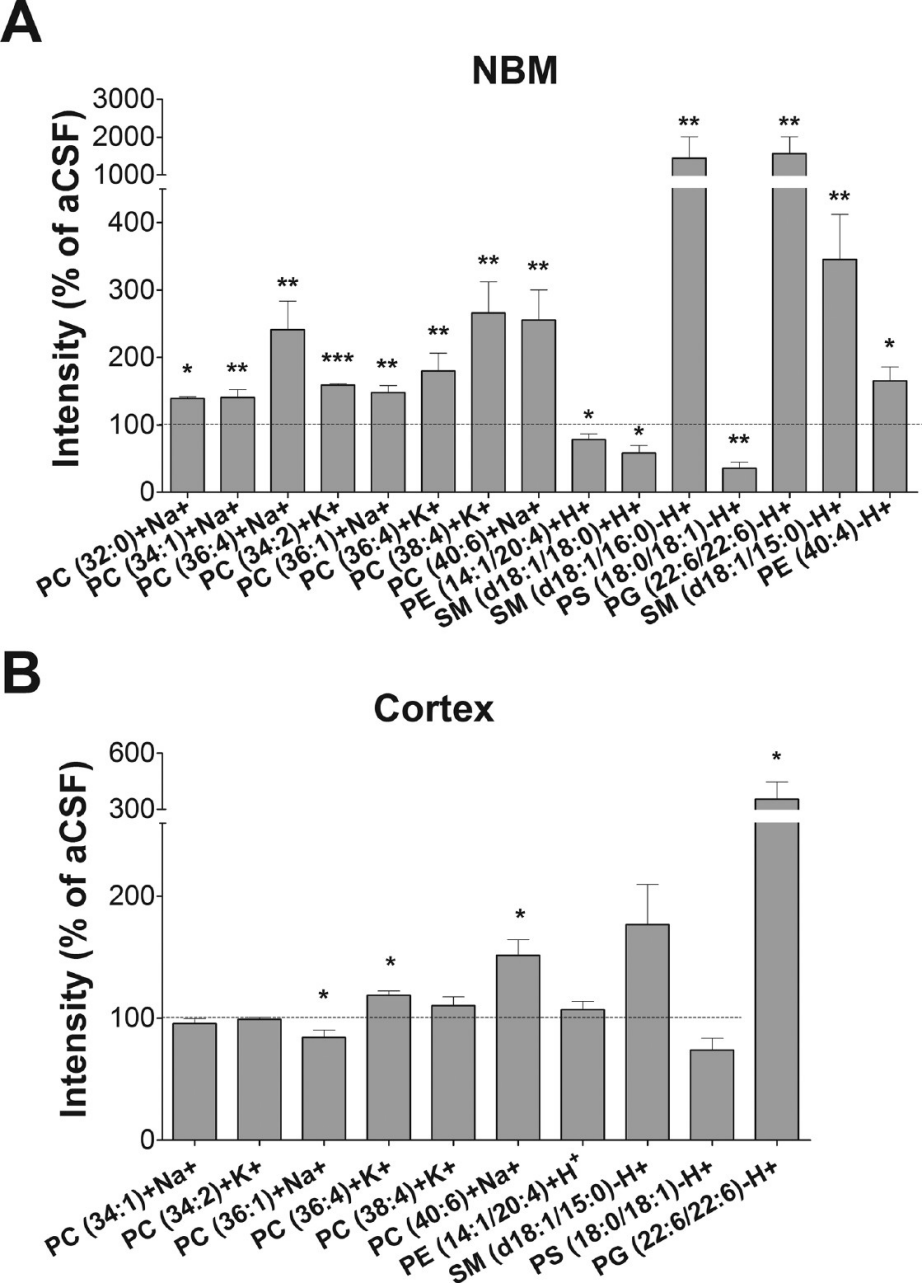
Relative abundance of different lipid species in NBM (A) and cortex (B), expressed as percentages of the control group values (aCSF): *p < 0.05, **p < 0.01, and ***p < 0.001, SAP vs aCSF-treated rats.

First, MALDI-IMS was performed in positive ion detection mode, and more than 300 peaks were obtained. There were changes in the relative levels of several phosphatidylcholine (PC) species in the NBM, but only three of them, PC (40:6) + Na^+^ (*p* < 0.01), PC (36:4) + K^+^ (*p* < 0.05), and PC (36:1) + Na^+^ (*p* < 0.01), were also regulated in the cortex of lesioned rats (Figures 2 and 3 and Supporting Information Table S1). However, a decrease of PC (36:1) + Na^+^ (*p* < 0.05) was found in the cortex. Although the precise physiological relevance of PC (36:1) + Na^+^ still remains unclear, its presence in rat brain and in myelinated regions in human brain has previously been described.^37−39^ Perhaps an incomplete removal of the BFCN debris and/or conversely a partial remodeling process in an attempt to promote axonal sprouting may explain the increase of this particular species in the NBM and the decrease in the cortex.

**Figure 4:**
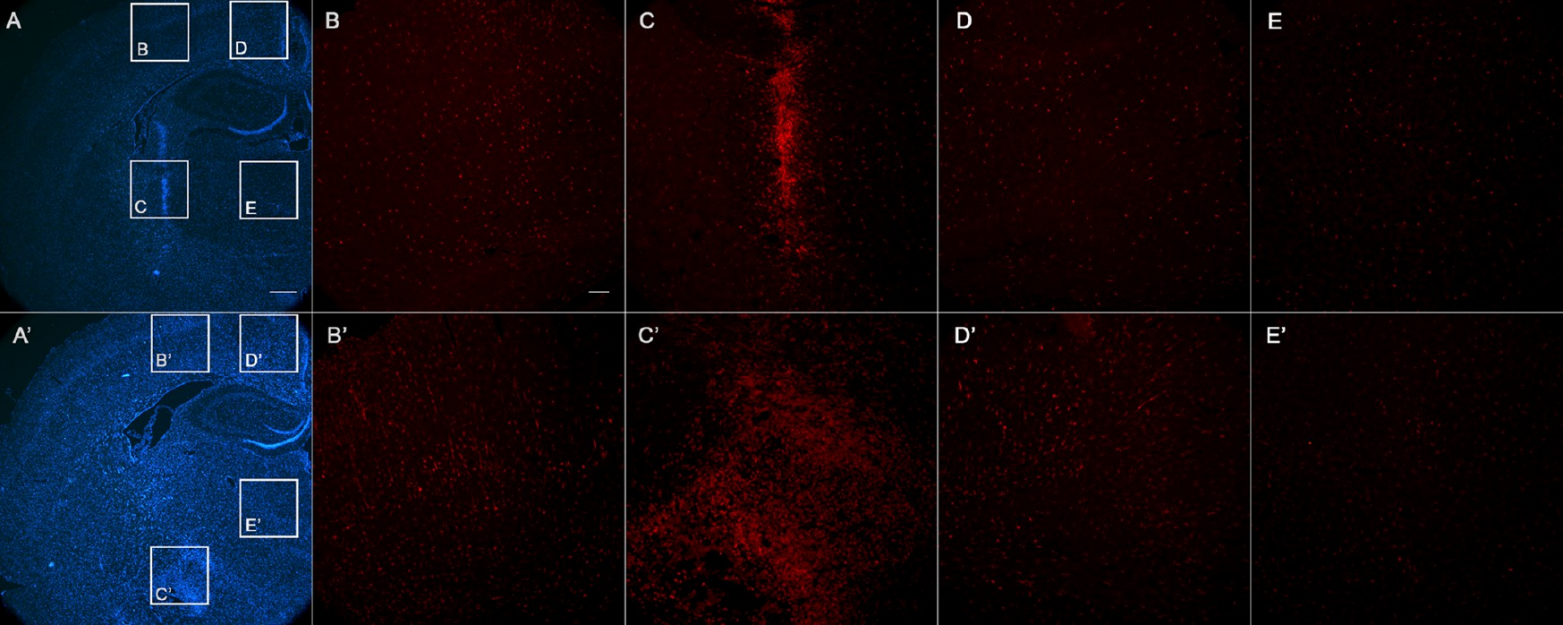
Hoechst staining (blue) and Iba1 (red), showing coronal representative sections of rat brains in the proximity of the injection site: (A, B, C, D, E) aCSF; (A′, B′, C′, D′, E′) SAP-treated. The inset squares indicate the brain areas where the microglia density is shown: (B, B′) cortical area near the injection; (C, C′) correspondence to the injection site at the NBM nucleus; (D, D′) cingulate cortex; (E, E′) area of thalamic nuclei. Note the higher density of Iba-1 positive cells (microglia) in lesioned animal, which is more noticeable and extended at the lesion site in the NBM. Bar in (A) is 500 μm. Bar in (B) is 100 μm.

On the other hand, some lipid species were clearly up-regulated after the lesion at both the NBM and cortex. PC (36:4) + K^+^, PC (38:4) + K^+^, and PC (40:6) + Na^+^, which are mainly present in gray matter,^30^ were increased in both areas. The most probable composition of PC (40:6) + Na^+^ in brain is stearic acid (18:0) and docosahexaenoic acid (DHA; 22:6), and of PC (36:4) + K^+^, palmitic acid (16:0) and arachidonic acid (AA; 20:4). AA and DHA are classical polyunsaturated fatty acids (PUFAs), which have been described to restore rat cerebral Ch and ACh levels, improving their behavioral outcomes in the PA test.^40,41^ The conversion of Ch to PC requires, among others, the combination with PUFAs such as DHA.^42^ In this regard, the density of PC (40:6) and other Ch-containing phospholipids has been found to be either increased^24,26^ or decreased^23,25^ in plasma samples from AD patients. The regulation of these specific lipid species has been proposed as a biomarker of phenoconversion to AD. The present results suggest that the general increase in these three different PCs, induced after the cholinergic lesion of BFCN, could represent a first response to the immunotoxin-induced massive death of BFCN in the NBM, since neuroinflammatory processes could still be present 7 days after lesioning, as indicated by the observed general increase in Iba-1 immunostaining (microglial-specific marker). The microglial proliferation showed a general increase in most of the brain areas following the immunotoxin administration. Moreover, the labeling was very high along the tract of the injection, especially in the cortex and NBM (Figure 4).

Other non-PC lipids were also found to be modified in the NBM, such as reduced levels of phosphatidylethanolamines (PEs) (14:1/20:4) + H^+^ and sphingomyelins (SMs) (d18:1/18:0) + H^+^ (*p* < 0.05) and the increase of SM (d18:1/16:0) + K^+^ (*p* < 0.01). Interestingly, the decreased levels of PE (14:1/20:4) in the NBM could reveal a process of autophagy^43^ or conversely be indicating a possible source for *de novo* synthesis of Ch.^44^ PEs from any membrane pool are sequentially methylated to PC by phosphatidylethanolamine *N*-methyltransferase and hydrolyzed to free Ch, a process that can be blocked by the mAChR antagonist atropine.^12,45−47^ Moreover, the stimulation of cortical synaptosomes with cholinergic agonists (e.g., ACh, muscarine or carbachol) is able to increase phospholipase D (PLD) activity, which is reduced by up to 63% in samples from AD patients.^48^ Then, mAChR-mediated signaling may be involved in the observed regulation of phospholipids after the BFCN lesion, pointing to PE (14:1/20:4) as another possible precursor for *de novo* synthesis of Ch. Furthermore, the negative ion detection mode revealed modifications in the levels of several additional lipid species in the NBM, such as an increase of SM (d18:1/15:0) (*p* < 0.01) and PE (40:4) (*p* < 0.05) and a reduction of phosphatidylserines (PS) (18:0/18:1) (aCSF 11.04 ± 2.16% vs SAP 3.95 ± 0.99%, *p* < 0.01). However, the level of only one species of phosphoglycerol (PG), PG (22:6/22:6), was increased in the group of SAP-treated rats in NBM and cortex (*p* < 0.05) (Figures 2 and 3, Supporting Information Table S1). Under normal physiological conditions, PS (18:0/18:1) is preferentially located in the inner leaflet of the plasma membrane. The reduction of this species and the loss of asymmetry, also described in AD patients, could represent an early indicator of apoptosis and/or glia-mediated synaptic pruning to remodel the damaged basal forebrain neural circuit.^49−51^

The changes in SM are discussed below. On the one hand, a significant increase in the relative intensity of two species, SM (d18:1/15:0) and SM (d18:1/16:0) + K^+^, was found in the NBM. On the other hand, reduced levels of SM (d18:1/18:0) + H^+^ revealed the loss of this particular species following the cholinergic lesion. Sphingolipid metabolism is essential for tissue homeostasis, and the catabolism of sphingolipids contributes to AD pathology.^52,53^ Interestingly, SM is characterized by carrying a Ch in their molecular structure and it is tempting to hypothesize that following the BFCN lesion, SM could be exploited to maintain cortical cholinergic neurotransmission when approximately 80% of the cortical cholinergic input has disappeared. Since Ch is not stored in vesicles, the generation of pools of lipids containing Ch for the further synthesis of ACh is difficult to understand, and one would expect to observe a reduction in any possible SM species for Ch synthesis, pointing to SM (d18:1/18:0) + H^+^ as a plausible candidate. However, the increase in the relative density of a specific SM species such as (d18:1/16:0) + K^+^ additionally allows us to hypothesize that a possible reservoir is created by an unknown storing mechanism from which to further obtain Ch. In AD patients, a depletion of SM (d18:1/16:0) in brain-derived nanoparticles fraction of CSF and a reduction in acid sphingomyelinase activity have been reported,^54^ whereas a marked increase in SM (d18:1/18:0) has been found in the hippocampal gray matter and in the CSF.^55,56^ Nevertheless, as the IMS technique allowed us to anatomically localize the specific up-regulation of these three lipids along the tract of the needle used for the lesion, we observed that it was coincident with the microglial response and we do not rule out the possibility that this could additionally explain the observed changes in distribution of these SM species.

### Altered Functional Coupling and Distribution of mAChR Following Basal Forebrain Cholinergic Depletion

**Figure 5:**
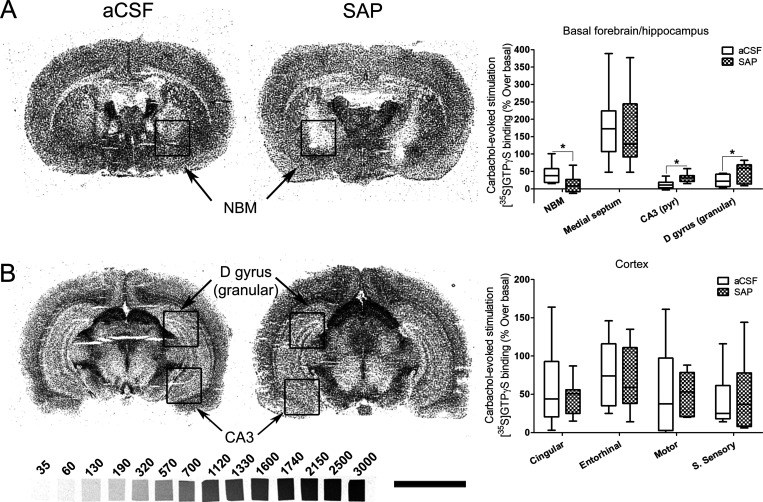
[^35^S]GTPγS autoradiography in rat brain coronal sections at two different levels from Bregma, (A) including NBM and (B) including dorsal hippocampus, obtained from aCSF, left (*n* = 9), and SAP-treated rats, right (*n* = 11), that show representative autoradiograms of [^35^S]GTPγS binding evoked by carbachol (100 μM). This assay is specific to G_i/o_ coupled receptors; therefore we are measuring the activity mediated by M_2_/M_4_ mAChR. The graphs show the mean ± SEM of each group in the different analyzed areas. NBM: nucleus basalis magnocellularis. D gyrus (granular): granular dentate gyrus. CA3: CA3 region of hippocampus. S sensory: somatosensory cortex. [^14^C]-Microscales were used as standards in nCi/g t.e. Scale bar: 5 mm.

**Figure 6:**
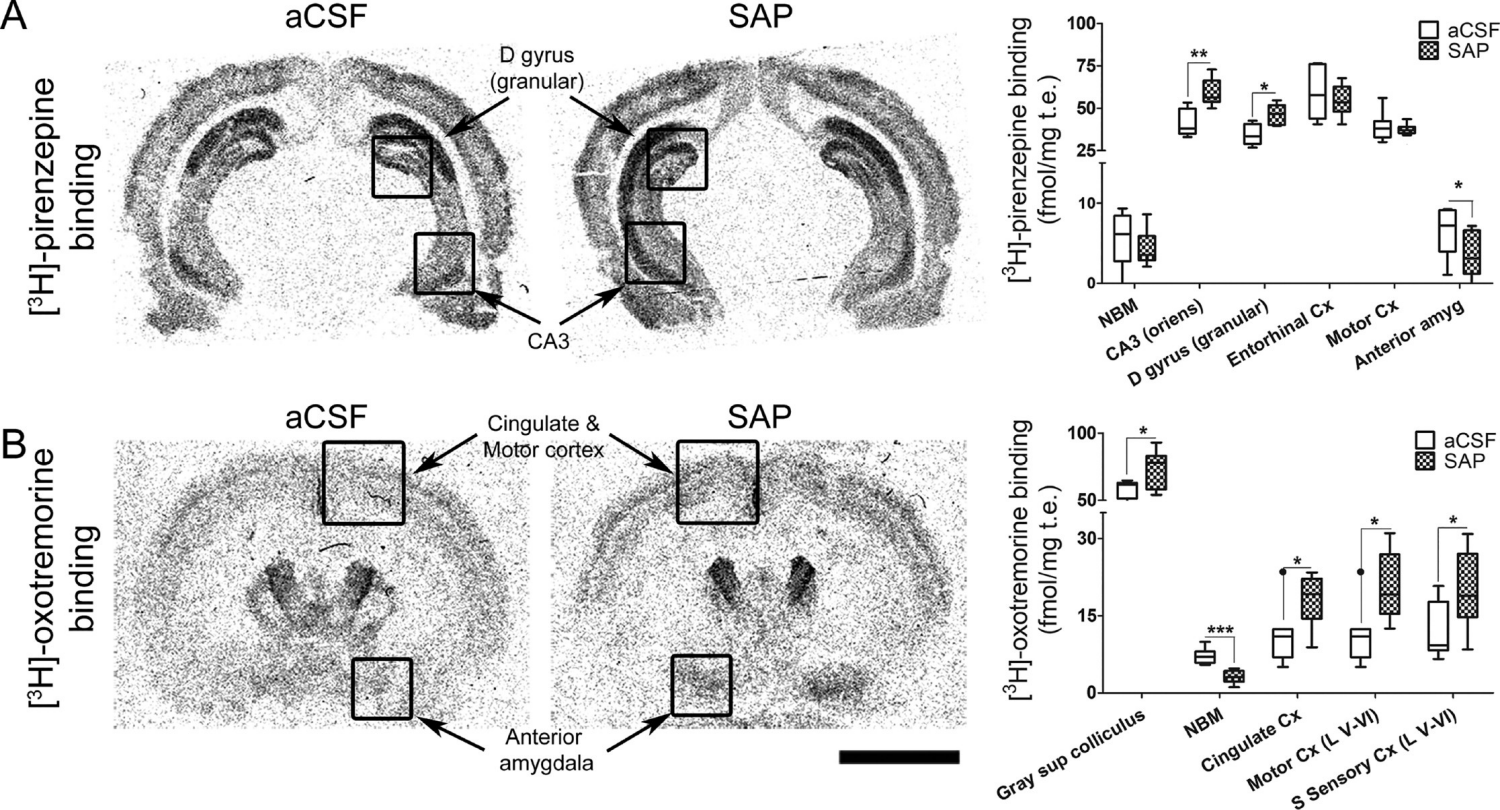
[^3^H]-Pirenzepine binding (A) and [^3^H]-oxotremorine binding (B) in rat brain coronal sections obtained from aCSF (*n* = 7) and SAP-treated rats (*n* = 9) that show the specific distribution of M_1_ mAChR and M_2_ mAChR, respectively. Note the increase of both the M_1_ mAChR density in the hippocampus (A) and the M_2_ mAChR density in the deepest layers of cortex (B) and in the anterior amygdala of SAP-treated rats. Note also the loss of both M_1_ mAChR in the anterior amygdala (A) and M_2_ mAChR in the NBM (B) following the lesion. The histograms show the mean ± SEM of each group in the different areas analyzed. Scale bar: 5 mm.

**Figure 7:**
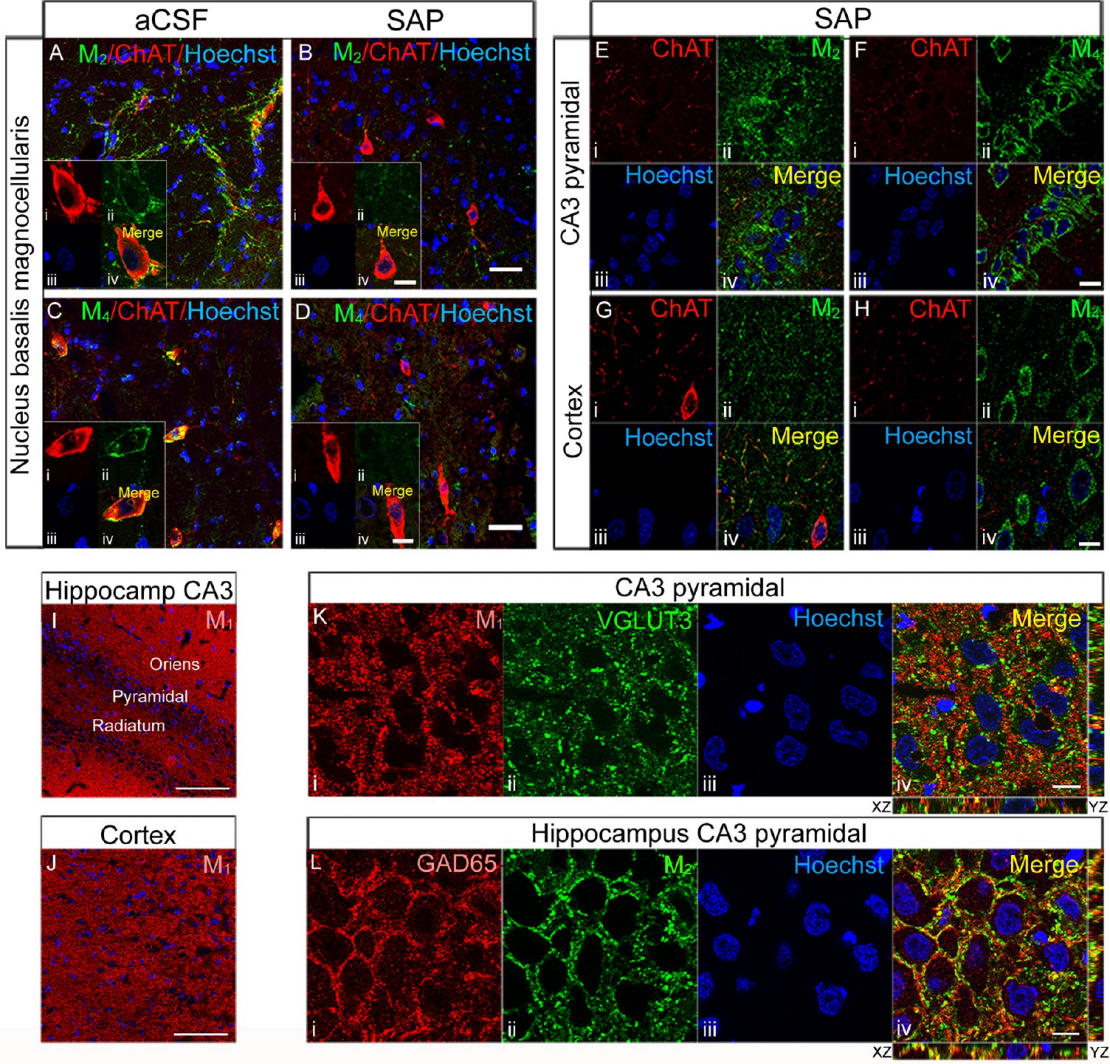
Double labeling in sections containing NBM from aCSF (A, C) and 192IgG-saporin-treated (B, D) rats, stained for ChAT (red) and M_2_ mAChR and M_4_ mAChR (green) at 200-fold magnification. 192IgG-saporin induced a reduction in BFCN density and in M_2_/M_4_ mAChR-immunoreactivity. Scale bars (B, D) = 40 μm. High magnification images show M_2_ mAChR immunoreactivity surrounding the perikarya (Aii) of the large BFCN (Ai) with a modest degree of colocalization (Aiv), whereas M_4_ mAChR-immunolabeling, surrounding the perikarya (Cii) of the large BFCN (Ci), shows a high degree of colocalization (Civ). Interestingly, 192IgG-saporin-treated rats show the presence of shrunk ChAT-immunoreactive neurons (Bi, Di), where the loss of M_2_ and M_4_ mAChR immunoreactivity is evident (Bii and Dii, respectively). Scale bars (Biv, Div) = 10 μm. Double labeling of consecutive sections containing hippocampal CA3 pyramidal region (E, F) and somatosensory cortex (G, H) from one SAP-treated rat, stained for ChAT (red), M_2_ mAChR (E and G in green), and M_4_ mAChR (F and H in green) at 630-fold magnification. The images show a presynaptic distribution of M_2_ mAChR, delineating the perikarya of the large CA3 pyramidal neurons in basket-like formations (Eii) and the somatodendritic distribution of M_4_ mAChR in the perikarya of the same neurons (Fii). In the cortex, both M_2_ (G) and M_4_ (H) mAChR distribution displays a similar pattern to that observed in CA3. Scale bars (Fiv, Hiv) = 10 μm. M_1_ mAChR immunolabeling in hippocampal CA3 region (I) and somatosensory cortex (J) from one SAP-treated rat at 200-fold magnification. Scale bars (I, J) = 100 μm. 630-fold magnification images show M_1_ mAChR (*K*_i_) immunoreactivity distributed surrounding the nuclei (Kiii) in the perikarya of pyramidal neurons with a modest degree of colocalization (Kiv) with the glutamatergic marker VGLUT3 (Kii). M_2_ mAChRs (Lii) are distributed in presynaptic GABAergic terminals (Li) in basket-like formations surrounding the somatodendritic compartment of pyramidal neurons of CA3 with a high degree of colocalization (Liv). Scale bars = 10 μm.

In an attempt to relate the changes in the lipid distribution and cholinergic muscarinic signaling, exhaustive autoradiographic and immunohistochemical studies were carried out. To examine M_2_/M_4_ mAChR-mediated signaling, the activity of G_i/o_-coupled mAChR was rostrocaudally measured from the medial septum to the ventral hippocampus by using [^35^S]GTPγS autoradiography assay (Figure 5, Supporting Information Tables S2–S4). The anatomical distribution and quantification of M_1_ mAChR and M_2_ mAChR were performed using [^3^H]-pirenzepine in the presence of unlabeled oxotremorine and [^3^H]-oxotremorine in the presence of pirenzepine, respectively, showing the characteristic patterns of distribution, i.e., M_1_ mAChR abundant in the cortical mantle, hippocampus, basal ganglia, and amygdala, whereas M_2_ mAChRs are less abundantly distributed in cortical and hippocampal regions and more in the basal forebrain and in thalamic areas (Figure 6A,B, Supporting Information Table S5). To examine the cellular localization of mAChR, immunofluorescence studies were performed using an antiserum against i3 intercellular loop of human M_1_, M_2_, and M_4_ mAChR (Figure 7). The pattern of distribution of M_1_, M_2_, and M_4_ mAChR was very similar to that observed in the autoradiographic studies (Figure 6).

Following the lesion, the functionality of mAChR was found to be decreased in the NBM (*p* < 0.05), unaltered in cortical areas, and enhanced in the hippocampal pyramidal layer of the CA3 (*p* < 0.05) and in the granular layer of the dentate gyrus (*p* < 0.05) (Figure 5). In the cortex, despite the elimination of up to 70% of BFCN in some cortical regions (as measured in the AChE assay, Figure 1G), no differences in the functional coupling of M_2_/M_4_ mAChR to G_i/o_ proteins were observed. The mAChR autoradiographic studies revealed a loss of M_2_ mAChR in the NBM (*p* < 0.001), whereas increased levels were found in the superficial gray layer of superior colliculus (*p* < 0.05) as well as in several cortical regions (*p* < 0.05) (Figure 6B, Supporting Information Table S5). On the other hand, increased levels of M_1_ mAChR were found in the oriens layer of hippocampal CA3 and in the granular region of dentate gyrus (*p* < 0.01) but decreased levels in the anterior amygdala (*p* < 0.05) (Figure 6A, Supporting Information Table S5). BFCN of NBM displayed a modest somatodendritic immunostaining, but the presence of a dense network of fibers surrounding the somas revealed presynaptic contacts from M_2_ mAChR-positive axon terminals (Figure 7Eiv).

SAP-treated rats showed an almost total absence of M_2_ mAChR-immunoreactivity in the basal forebrain due to the near disappearance of BFCN, as revealed by ChAT immunostaining (Figure 7Biv). On the other hand, M_4_ mAChR-immunoreactivity was distributed mainly in somatodendritic compartments in NBM (Figure 7C,D), hippocampus and cortex (Figure 7Fii,Hii), displaying a typically postsynaptic localization. Immunotoxin-treated rats showed a dramatic decrease in M_4_ mAChR-immunoreactivity in the basal forebrain, accompanied by the disappearance of BFCN (Figure 7Dii). These findings demonstrate the contribution of these receptors to the cholinergic neurotransmission impairment and the loss of cholinergic interconnections in the basal forebrain. Although the precise anatomical distribution of M_2_ and M_4_ mAChR in the NBM remains controversial, the present immunohistochemical findings suggest a mainly presynaptic localization of M_2_ mAChR and a somatodendritic distribution of M_4_ mAChR. This specific localization is important to understand the physiological consequences of the lesion. In the somatosensory cortex, M_2_ mAChR presented a scattered distribution in presumably presynaptic compartments (Figure 7Gii). A high expression of postsynaptic M_1_ mAChR was found in the cortical mantle, the hippocampus, the amygdaloid complex, and striatum (Figure 7I,J). At the subcellular level, the mAChR subtypes were differentially distributed in the CA3 region of the hippocampus. M_1_ mAChR (Figure 7Kiv) and M_4_ mAChR (not shown) were distributed in glutamatergic postsynaptic compartments, while a high degree of colocalization between M_2_ mAChR and the presynaptic GABAergic marker GAD65 was observed (Figure 7Liv). M_2_ mAChR-immunoreactivity was differentially distributed throughout the hippocampal formation. The large pyramidal neurons from CA1–CA3 and dentate gyrus displayed a dense network of fibers, which delineate the perikarya in basket-like formations, without immunoreactivity in the soma (Figure 7Eiv,Lii). In the granular dentate gyrus and pyramidal CA1–CA3, M_2_ mAChRs are located in inhibitory GABAergic presynaptic terminals, whereas M_1_ and M_4_ mAChRs are distributed in the somatodendritic compartment of pyramidal neurons. The increase in cortical M_2_ mAChR may indicate a secondary regulation of cortical M_2_ mAChR, which is consistent with previous studies.^16,57−61^ Regarding the modulation of hippocampal cholinergic signaling, lesions directed at the medial septum-vertical diagonal band complex have demonstrated a decrease in both density and activity of mAChR, revealing completely opposite effects to those observed when the NBM is lesioned.^62−66^ The absence of nonspecific damage to the SM-vertical diagonal band complex indicates that the increase in M_2_/M_4_ mAChR-mediated signaling in the hippocampus should also be considered as a secondary modulation of muscarinic signaling. In the hippocampus, M_2_ mAChR inhibits ACh release, while M_1_ mAChR potentiates glutamatergic signaling.^65,67−69^ Moreover, mice lacking M_2_ mAChR showed deficits in learning during avoidance and spatial memory tests, demonstrating the role of this receptor subtype in cognitive processes which are dependent on both short- and long-term potentiation and completely reversed with GABA_A_ receptor antagonists.^70,71^ Therefore, the immunotoxin-induced increase in hippocampal muscarinic functionality mediated by M_2_ mAChR could be decreasing the GABAergic tone and increasing glutamatergic transmission as a compensatory mechanism following the lesion of the NBM, contributing to the so-called “muscarinic long-term potentiation”, which is essential to explain hippocampal neuronal plasticity.^72^

### Muscarinic Receptor-Mediated Signaling Induces Specific Changes in the Lipid Profile in *ex Vivo* Organotypic Cultures

**Figure 8:**
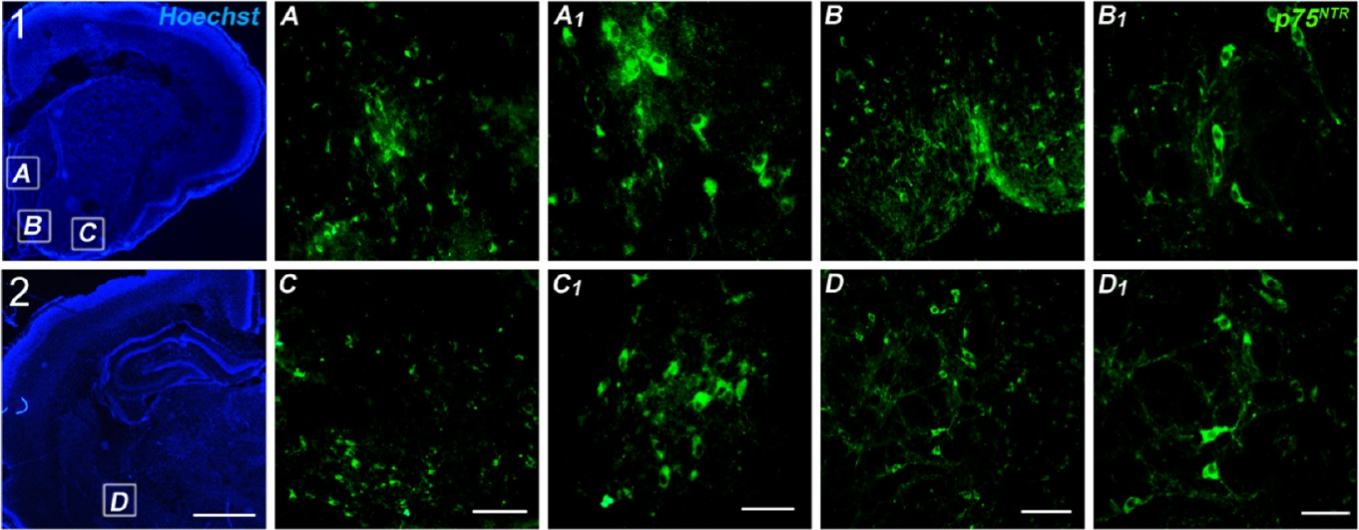
Low magnification (2.5×) photomicrographs of Hoechst staining in 10 μm tissue slices from P7 rats including the medial septum (MS) (1A), both vertical diagonal band of Broca (1B) and horizontal diagonal band of Broca (1C) and the NBM (2D) (scale bar = 1 mm). P75^NTR^ immunofluorescence in the MS (A, A_1_). Vertical diagonal band (B, B_1_). Horizontal diagonal band (C, C_1_). NBM (D, D_1_). (C, D) Scale bar = 100 μm. (C_1_, D_1_) Scale bar = 50 μm.

The MALDI-IMS technique was also used to semiquantify the relative intensity of several lipids after specific mAChR stimulation in *ex vivo* organotypic cultures. The immunofluorescence studies carried out in the basal forebrain cholinergic system from P7 rats revealed the presence of a high density of BFCN expressing p75NTR supporting the rationale of using this model to further investigate the modulation of lipid composition mediated by muscarinic signaling (Figure 8). *Ex vivo* organotypic cultures including the BFCN and cortical regions were used to analyze the lipid modifications following treatment with the muscarinic agonist carbachol or with the nonselective antagonist scopolamine and the M_1_ selective pirenzepine.

**Figure 9:**
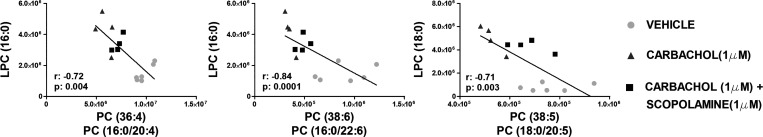
Correlation analyses between PC 36:4, PC 38:6, and PC 38:5 with LPC 16:0 and 18:0 in organotypic cultures following three different experimental treatments (vehicle, carbachol 1 μM, and carbachol 1 μM + scopolamine 1 μM): *p < 0.05, **p < 0.01, and ***p < 0.001.

We found decreased levels of several PC induced by carbachol (e.g., 36:4, O-36:4, 36:5, 38:5, 38:6, 38:7, O-38:7, and 40:7+ H^+^) (Supporting Information Table S6). Different pathways related to muscarinic signaling could govern the metabolism of PC including the following: phospholipase C-mediated degradation leading to formation of diacylglycerol (DAG) and phosphocholine or phospholipase D catalyzing the hydrolysis to phosphatidic acid (PA) and Ch.^73−76^ However, we only found increased lysophosphatidylcholines (LPCs) (16:0, 16:1, 18:0, 18:1, and 18:2) + H^+^ and LPC (O-18:0) −CH_3_. LPC species are derived by the cleaving of PC via the action of phospholipase A2 (PLA2) hydrolyzing the fatty acid at position 2 of phospholipids, generating free fatty acid (DHA or AA).^77,78^ More studies are needed to completely discard the action of phospholipase C or PLD activation, since the absence of an increase of DAG and PA levels in organotypic cultures could be a result of the downstream degradation in their signaling pathways. Conversely, the negative correlation between the reduction of PC and the increase of LPC supports the PLA2 action by mAChR activation (Figure 9). In addition, the modulation of PC and LPC species after carbachol administration is abolished by scopolamine but not with pirenzepine, suggesting that this effect is not dependent on M_1_ mAChR. In this sense, several studies support the involvement of PLA2 in the production of free Ch for the synthesis of ACh, which is found coupled to mAChR.^79,80^ As previously shown, decreased muscarinic activity in NBM after the lesion could be related to the increase of PC in NBM by a PLA2 down-regulation. Finally, common lipid species (PC 36:4, 38:7, and 38:4 + H^+^) were found to be increased *in vivo* following cholinergic depletion and decreased in *ex vivo* organotypic cultures after mAChR activation. All the results indicate that these PC species could be possible lipid precursors of ACh in this specific pathway.

The dramatic reduction of AChE staining in basal forebrain and projections to cortical areas, together with the observed learning and memory impairment, validates the use of this model of NBM lesion in rat for the study of possible adaptations of the lipid profile in relation to cholinergic neurotransmission impairment. The loss of most of the BFCN shortened the ACh supply, but part of these cells, which continue to be physiologically active, could make use of specific membrane PL precursors of Ch as an emergency source for obtaining the cholinergic neurotransmitter. The reduction in phospholipid levels could lead to a decrease of membrane reservoirs available for different purposes including dendritic spinogenesis for new synapses after long-term potentiation processes (learning). In fact, we observed shrinkage of the surviving BFCN. The hydrolysis of PL from membranes of presynaptic terminals (cortical area) and/or somatodendritic compartment (NBM area) may be responsible for the selective regulation of certain lipid species following the lesion that could be controlled by muscarinic signaling. MALDI-IMS analysis showed changes in the lipid profile, which include some specific species of PC, PE, PS, and SM. Despite the abundance of M_1_ mAChR in cortical regions, both autoradiographic and immunohistochemical results failed to show changes induced by the cholinergic lesion, which suggests that M_1_ mAChR-mediated signaling may not be related to the observed lipid modulation. Thus, the observed modulation of the lipidome by the cholinergic metabolism is probably mediated mainly by those mAChR coupled to G_i/o_ proteins (i.e., M_2_ and M_4_ subtypes). Possible choline precursors such as some PC species, are increased in the rat lesion model at the NBM, where the M_2_ mAChR density and activity are decreased, diminishing the PLA2 activity that results in higher PC levels. Conversely, the M_2/4_ mAChRs are up-regulated in the cortex of lesioned rats and consequently the PC intensities.

Altogether the above-described processes indicate the control of PC metabolism by M_2/4_ mAChR when the availability of ACh is compromised.

## Conclusions

In summary, the loss of cortical cholinergic innervation leads to learning and memory impairment involving the modulation of the activity of M_2_/M_4_ mAChR in the NBM and hippocampus but not in the cortex, despite the fact that increased densities of M_2_ mAChR were recorded in several cortical regions. Moreover, an up-regulation of M_1_ mAChR was observed in the hippocampus together with changes in M_2_/M_4_ mAChR activity in this specific brain region, revealing a secondary modulation of muscarinic signaling. The present findings show up-regulated lipid levels indicating not only a first microglial response but also the specific cortical down-regulation of potential Ch precursors as an alternative source for ACh. This regulation may not be mediated through M_1_ mAChR. However, we cannot rule out the possibility of a G_i/o_-coupled mAChR-mediated (i.e., M_2_ mAChR) modulation of the lipid composition. The present results contribute to the understanding of the brain adaptations involving microglial response, possible *de novo* synthesis of Ch and the mAChR-mediated regulation of lipid metabolic pathways following the specific loss of BFCN and cholinergic input. The AD rat model used in the present study provides us with a suitable tool with which to obtain further knowledge about the relationship between ACh metabolism and muscarinic signaling. Future treatments for AD based on potentiating cholinergic neurotransmission could involve the regulation of the lipid metabolism through modulation of specific mAChR subtypes.

## Methods

### Chemicals

192IgG-saporin (batch 2441969) was acquired from Millipore (Temecula, CA, USA). RRID AB_94979, [^3^H]-pirenzepine (86.0 Ci/mmol, catalog no. NET80250UC), [^3^H]-oxotremorine (75.8 Ci/mmol, catalog no. NET671), and [^35^S]GTPγS (1250 Ci/mmol, catalog no. NEG030H250UC) were from PerkinElmer (Boston, MA, USA). Both [^3^H]- and [^14^C]-microscales (catalog no. ART0123A and catalog no. ARC0146, respectively) were used as standards in the autoradiographic experiments, ARC (American Radiolabeled Chemicals, Saint Louis, MO, USA). The β-radiation-sensitive Kodak Biomax MR films (catalog no. 7358460), bovine serum albumin (BSA) (catalog no. A4503), carbachol (catalog no. C4382), pirenzepine (catalog no. P7412), oxotremorine (catalog no. O9126), atropine (catalog no. A0257) scopolamine (catalog no. S0929), dl-dithiothreitol (DTT) (catalog no. D5545), adenosine deaminase (ADA) (catalog no. A9876), guanosine 5′-diphosphate (GDP) (catalog no. G7127), guanosine 5′-*O*-3-thiotriphosphate (GTPγS) (catalog no. G8634), ketamine (catalog no. K2753), xylazine (catalog no. X1251), acetylthiocholine iodide (catalog no. 01480), 2-mercaptobenzothiazole (MBT) (catalog no. M3302), and tetraisopropyl pyrophosphoramide (iso-OMPA) (catalog no. T1505) were all acquired from Sigma-Aldrich (St. Louis, MO, USA).

### Animals

Ninety-seven adult male Sprague-Dawley rats, weighing 225–275 g and ranging in age from 8 to 10 weeks, were used in this study. Rats were housed four per cage (50 cm, length × 25 cm, width × 15 cm, height) at a temperature of 22 °C in a humidity-controlled (65%) room with a 12:12 h light/dark cycle with access to food and water ad libitum. Seven rat pups at postnatal day 7 (P7) weighed 14–20 g at the start of the *ex vivo* experiments based on hemibrain organotypic cultures containing BFCN.

All procedures were performed in accordance with European animal research laws (Directive 2010/63/EU) and the Spanish National Guidelines for Animal Experimentation (RD 53/2013, Law 32/2007). Experimental protocols were approved by the Local Ethics Committee for Animal Research of the University of the Basque Country (CEEA 388/2014). The authors further attest that all efforts were made to minimize the number of animals used and their suffering.

### *Ex Vivo* Organotypic Cultures

To prepare hemibrain organotypic cultures, P7 Sprague-Dawley rats (*n* = 7) were sacrificed by decapitation and their brains were quickly dissected under aseptic conditions inside a laminar flow cabinet (TELSTAR, BV-30/70).^81^ After removal of the olfactory bulb and the most caudal part of the cerebellum, the brains were placed in minimal essential Dulbecco′s modified Eagle medium (DMEM, Sigma-Aldrich) supplemented with 0.1% (v/v) antibiotic/antimycotic (Gibco) at 4 °C.

The brains were vertically positioned resting on the cerebellum by means of cyanoacrylate. They were then cut, from the rostral to the caudal part, into coronal 300 μm thick organotypic slices using a sliding vibratome (Leica VT 1.000 S, Leica Microsystems AG, Wetzlar, Germany). Approximately 6 slices containing the medial septum and another 6 slices containing the NBM were obtained from each brain, and these were immediately transferred into cell culture inserts over membranes of 0.4 μm pore size (PIC50ORG, Millipore, MA, USA). The organotypic slices were then placed in 6-well cell culture plate (Falcon, BD Biosciences Discovery Labware, Bedford, MA) that contained 1 mL of culture medium per well. The culture medium consisted of 49% (v/v) neurobasal medium (NB, Sigma-Aldrich), 24% (v/v) Hanks’ balanced salt solution (HBSS, Gibco), 24% (v/v) normal horse serum (NHS, Gibco), 1% (v/v) d-glucose, 0.5% glutamine (Sigma-Aldrich), 0.5% B27 supplement serum free (Gibco), and 1% antibiotic/antimycotic. The culture plates were incubated at 37 °C in a fully humidified atmosphere supplemented with 5% CO_2_, and the cell culture medium was replaced by fresh medium on the second day.

### Basal Forebrain Cholinergic Lesion

All surgery was carried out under aseptic conditions. 192IgG-saporin was used to selectively eliminate BFCN in the NBM. Rats were randomly assigned to one of three groups: sham-operated (SHAM; *n* = 6), artificial cerebrospinal fluid, used as vehicle (aCSF; *n* = 44), and 192IgG-saporin (SAP; *n* = 47). SHAM was the first group analyzed. Then, aCSF- and SAP-treated animals were further assessed in five independent groups (aCSF, *n* = 9; SAP, *n* = 9). The vehicle was prepared as follows: 0.15 M NaCl, 2.7 mM KCl, 0.85 mM MgCl_2_, 1.2 mM CaCl_2_ (pH 7.4) and sterilized by filtration with 0.4 μm ⌀ filters (EMD Millipore, CA, USA). The intraparenchymal infusion of aCSF or SAP was made into the NBM: −1.5 mm anteroposterior from Bregma, ±3 mm mediolateral from midline, +8 mm dorsoventral from cranial surface,^82^ as previously described.^17^ Rats were kept warm and hydrated and continuously monitored during surgery. Following surgery, they were kept away from other animals until fully recovered.

### Passive Avoidance Test

The rats were allowed seven complete days to recover from surgery and were then subjected to the passive avoidance test (PanLab passive avoidance box LE870/872). The passive avoidance test involves two sessions. The first, which is called “the learning trial”, was performed on day 7 after surgery beginning at 8 a.m. Each animal (SHAM, *n* = 6; aCSF, *n* = 36; SAP, *n* = 36) was gently placed in the illuminated compartment and allowed to explore it for 30 s. Then, the guillotine door was automatically opened and the rat was given a maximum of 60 s to enter the dark compartment. When the rat crossed the doorway the door closed, the acquisition latency time (i.e., the time that the rats remained in the open-topped compartment) was measured, and a foot shock (0.4 mA/2 s) was delivered. 10 s after the foot shock, the rat was returned to its home cage. 24 h later beginning at 8 a.m., for the second session called “the retention trial”, the rats were again placed in the illuminated compartment and allowed to explore for 30 s. Then the door opened and the step-through latency time (i.e., time taken to enter the dark compartment), was measured, with a cutoff of 300 s. Another group of rats (*n* = 19; aCSF, *n* = 8; SAP, *n* = 11) was used to record the electric-shock-evoked first vocalization by two observers when delivering increasing foot shock intensities. Each animal was gently placed in the illuminated compartment and allowed to explore it for 30 s. When the rat crossed the doorway, increasing intensities of 0.05 mA lapses (1 s duration) and ranging from 0.0 mA to 0.4 mA were given, and the minimum electrical intensity for the first audible vocalization was recorded. These animals were discarded from further behavioral studies.

### Tissue Preparation

Following the passive avoidance test, all the animals were anesthetized with ketamine/xylazine (90/10 mg/kg; ip) and killed by decapitation or transcardially perfused to obtain fresh or fixed tissue, respectively.

#### Fresh Tissue

The brains from aCSF (*n* = 31) and SAP (*n* = 32) groups were quickly removed by dissection, fresh frozen, and kept at −80 °C. Later, they were cut into 20 μm sections using a Microm HM550 cryostat (Thermo Scientific) equipped with a freezing-sliding microtome at −25 °C and mounted onto gelatin-coated slides and stored at −25 °C until used.

#### Fixed Tissue

Representative animals from aCSF and SAP groups, which showed a positive or negative response respectively in the passive avoidance test, were transcardially perfused with 50 mL of warm (37 °C), calcium-free Tyrode’s solution (0.15 M NaCl, 5 mM KCl, 1.5 mM MgCl_2_, 1 mM MgSO_4_, 1.5 mM NaH_2_PO_4_, 5.5 mM glucose, 25 mM NaHCO_3_; pH 7.4), 0.5% heparinized, followed by 4% paraformaldehyde and 3% picric acid in 0.1 M phosphate buffer (PB) (4 °C) (100 mL/100 g, bw) (37 °C, pH 7.4). The brains were removed, followed by immersion in a cryoprotective solution of 20% sucrose in PB overnight at 4 °C, and then frozen by immersion in isopentane and kept at −80 °C. Later they were coronally cut into 10 μm sections as described above and mounted onto gelatin-coated slides and finally stored at −25 °C until use for the immunofluorescence assays.

After 2 days *in vitro*, organotypic cultures were treated with vehicle (0.9% saline solution), carbachol (1 μM), carbachol (1 μM) and scopolamine (1 μM) or carbachol (1 μM) and pirenzepine (1 μM) for 72 h, respectively. Then, organotypic culture tissue from vehicle (*n* = 3), carbachol (*n* = 2), carbachol and scopolamine (*n* = 2) or carbachol and pirenzepine (*n* = 2) conditions were washed with 0.1 M PB. Lastly, samples were homogenized using a Potter-Elvehjem tissue homogenizer in water and centrifuged at 15 000 rpm for 15 min at 4 °C. The pellet was frozen at −80 °C until IMS experiment was carried out.

Other organotypic cultures with no treatment (*n* = 2) were used for immunohistochemical studies. They were rinsed with 0.9% saline solution (37 °C) followed by immersion in 4% paraformaldehyde and 3% picric acid in 0.1 M PB (4 °C) for 1 h. Then, the fixed organotypic slices were extensively rinsed in 0.1 M PB (pH 7.4).

### AChE Staining and Quantitative Analysis

Fresh tissue slices containing basal forebrain and hippocampus from aCSF (*n* = 13) and SAP (*n* = 13) treated rats were fixed in 4% paraformaldehyde in PB (0.1M) for 30 min at 4 °C and washed in 0.1 M PBS, pH 7.4, for 20 min. Cholinergic innervations were stained using the “direct coloring” thiocholine method for AChE^83^ as follows. The sections were rinsed twice in 0.1 M Tris-maleate buffer (pH 6.0) for 10 min and incubated for 100 min in complete darkness in the AChE reaction buffer (0.1 M Tris-maleate, 5 mM sodium citrate, 3 mM CuSO_4_, 0.1 mM iso-OMPA, 0.5 mM K_3_Fe(CN)_6_, and 2 mM acetylthiocholine iodide). The enzymatic reaction was stopped with two consecutive rinses (2 × 10 min) in 0.1 M Tris-maleate (pH 6.0). The sections were scanned at 600 ppi, and the images were converted to 8-bit gray scale mode. AChE positive fiber density was quantified using ImageJ software (NIH, Bethesda, MD, USA). The intensity values in arbitrary units were defined in the selected equivalent areas from both hemispheres. Background was subtracted from AChE positive signals to obtain the net AChE optical density in each area. AChE staining values in the striatum served as control, and data were expressed for each brain area as a percentage of striatal levels.

### [^35^S]GTPγS Autoradiography and Quantitative Analysis of Autoradiograms

The functional coupling of mAChR to G_i/o_ proteins was measured in fresh frozen 20 μm sections from each rat of both groups, aCSF (*n* = 9) and SAP (*n* = 11), as previously described.^33^ The slides were exposed to β-radiation-sensitive autoradiographic films in hermetically closed cassettes together with a set of [^14^C] standards to calibrate the images (gray densities). The films were developed after 48 h, scanned, and quantified by transforming optical densities into nCi/g tissue equivalent (nCi/g t.e.) using a calibration curve defined by the known values of the [^14^C] standards using ImageJ software (RRID SCR_003070). The specific binding for each area was calculated using consecutive sections as follows. Nonspecific binding values were subtracted from the values obtained in both agonist-stimulated and basal-stimulated conditions. Then, the net basal stimulation values were subtracted from agonist-stimulated values to obtain net agonist-stimulation ([agonist-stimulated – nonspecific] – [basal – nonspecific]). The percentages of carbachol-evoked stimulation were calculated according to the following formula: ([^35^S]GTPγS agonist-stimulated binding × 100/[^35^S]GTPγS basal binding) – 100.

### Autoradiographic Labeling and Quantification of M_1_ mAChR and M_2_ mAChR by Using [^3^H]-Pirenzepine and [^3^H]-Oxotremorine

Fresh 20 μm brain sections from aCSF (*n* = 7) and SAP (*n* = 9) groups were dried for 10 min and submerged at room temperature (RT) for 15 min in Krebs buffer (11 mM NaCl, 5 mM KCl, 2.5 mM CaCl_2_, 1.18 mM MgSO_4_·7H_2_O, 25 mM NaHCO_3_, 5.5 mM glucose, 1.18 mM KH_2_PO_4_; pH 7.4) for M_1_ mAChR or in 20 mM HEPES buffer for M_2_ mAChR. Then, the incubation was performed at RT for 1 h in Krebs buffer in the presence of [^3^H]-pirenzepine (3 nM) and nonlabeled oxotremorine (300 nM) for labeling M_1_ mAChR or for 40 min in HEPES supplemented with [^3^H]-oxotremorine (2 nM) and nonlabeled pirenzepine (100 nM) for labeling M_2_ mAChR. In both cases, the nonspecific binding was measured by competition with nonlabeled atropine (10 μM) in another consecutive slice. Then, sections were washed in ice-cold (4 °C) 50 mM Tris-HCl buffer (pH 7.4) to stop the binding, dipped in distilled ice-cold water, and dried (4 °C). Autoradiograms were generated by exposure of the tissues for 35 days ([^3^H]-oxotremorine) or 52 days ([^3^H]-pirenzepine) at 4 °C to β-radiation-sensitive films in hermetically closed cassettes together with [^3^H]-microscales, which were used to calibrate the optical densities to fmol/mg tissue equivalent (fmol/mg).

### Immunofluorescence Studies

In order to perform the immunofluorescence assays, the frozen fixed tissue sections were previously dried at RT for 20 min. Anatomical distribution of BFCN was studied using goat anti-choline acetyltransferase (ChAT) [1:200] (RRID AB_2079751) as primary antiserum. To study the cellular localization of mAChR, primary rabbit anti-M_1_ mAChR [1:400] (RRID AB_2260554), rabbit anti-M_2_ mAChR [1:400] (RRID AB_2080068), mouse anti-M_4_ mAChR [1:250] (RRID AB_2080217), guinea pig antivesicular glutamate transporter 3 (VGLUT 3) [1:500] (RRID AB_2187832), and mouse antiglutamic acid decarboxylase isoform 65 kDa (GAD65) [1:750] (RRID AB_2263126) (EMD Millipore, CA, USA) were used. To detect microglial cells, primary rabbit polyclonal anti Iba-1 [1:1000] (Fujifilm Wako Chemicals, USA) was used. Primary antibodies were diluted in PBS (0.1 M, pH 7.4) containing 0.5% BSA, and the tissue samples were incubated overnight at 4 °C. They were then washed for 30 min in PBS and incubated for 30 min at 37 °C with the appropriate secondary antibodies. ChAT immunostaining was revealed with rhodamine-labeled donkey anti-goat [1:80] (RRID AB_2340388). M_1_ and M_2_ mAChR and Iba-1 were revealed with Alexa-fluor 488-labeled donkey anti-rabbit [1:250] (RRID AB_2535792) or CY3-labeled donkey anti-rabbit [1:250] (RRID AB_2307443). M_4_ mAChR and GAD65 were revealed with Alexa-fluor 488-labeled donkey anti-mouse [1:250] (RRID AB_141607), and VGLUT3 was revealed with Alexa-fluor 488-labeled donkey anti-guinea pig [1:250] (RRID AB_2340472). Then, the sections were incubated with Hoechst 33258 [1:106] (catalog no. B2883) for 15 min. Finally, sections were washed for 30 min by immersion in PBS and mounted with *p*-phenylenediamine-glycerol (0.1%) for immunofluorescence. 630-fold magnification images for colocalization (M_2_ mAChR-ChAT, M_4_ mAChR-ChAT, M_2_ mAChR-GAD65, and M_1_ mAChR-VGLUT3) were acquired on an Axioskop Observer A1 inverted microscope (Carl Zeiss) by optical sectioning (0.24 μm/*XYZ*-resolution) using structured illumination (ApoTome, Carl Zeiss). Colocalization images were created using ZEN2014 software (Carl Zeiss) and defined as immunosignals without physical signal separation.

Fixed organotypic slices were extensively rinsed in 0.1 M PB (pH 7.4) and simultaneously blocked and permeabilized with 4% normal goat serum (NGS) in 0.6% Triton X-100 in PBS (0.1 M, pH 7.4) for 2 h at 4 °C. All incubations were performed in free floating at 4 °C (48 h) with rabbit anti p75NTR primary antibody, diluted in 0.6% Triton X-100 in PBS with 5% BSA. The primary antibody was then revealed by incubation for 30 min at 37 °C with Alexa 488-labeled donkey anti-rabbit secondary antibody diluted in Triton X-100 (0.6%) in PBS. Then, organotypic slices were washed for 30 min by immersion in PBS and incubated for 15 min at room temperature with Hoechst 33258 for fluorescent counterstaining of the nuclei. Finally, slices were extensively rinsed with PBS and covered with *p*-phenylendiamine-glycerol (0.1%) in PBS for immunofluorescence observation.

### Sample Preparation for MALDI-IMS

Lipid composition and anatomical distribution were analyzed using MALDI-imaging mass spectrometry (IMS) in fresh 20 μm sections from each rat from the aCSF (*n* = 8) and SAP (*n* = 8) groups, following the recommendations of ref (84). Once the tissue was sliced and mounted on slides, MBT matrix was deposited on the tissue surface by sublimation. The sublimation was performed using 300 mg of MBT, and the deposition time and temperature were controlled (23 min, 100 °C). For the recrystallization of the matrix, the sample was attached to the bottom of a glass Petri dish face-down, which was placed on another Petri dish containing a methanol-impregnated piece of filter paper in its base. The Petri dish were then placed on a hot plate (1 min, 38 °C).

Then, lipid composition was analyzed in organotypic cultures. First, a dry pellet from organotypic cultures was obtained and the protein concentration was determined using the Bradford method.^85^ Samples were reconstituted with water at the same concentration. Then, a mixed sample (3 μL of sample and 7 μL of matrix-saturated solution of MBT) was deposited on MALDI plate containing 96 wells, using the dried droplet method.

### Mass Spectrometer and Image and Spectra Analysis

A MALDI LTQ-XL-Orbitrap (Thermo Fisher, San Jose, CA) equipped with a nitrogen laser (λ = 337 nm, rep rate = 60 Hz, spot size = 80 μm × 120 μm) was used for mass analysis. Thermo’s Imagequest and Xcalibur software were used for MALDI-IMS data and image acquisition in both positive and negative ion modes. The positive ion range was 500–1000 Da, and the negative ion range was 400–1100 Da, with 10 laser shots per pixel at a laser fluence of 15 μJ. The target plate stepping distance was set at 150 μm for both *x*- and *y*-axes by the MSI image acquisition software. The data were normalized using the total ion current values, as there may be potential displacement of the masses on the tissue due to experimental factors, e.g., the irregularities of the surface. The MALDI images were generated using ImageQuest (Thermo Scientific). Each of the *m*/*z* values was plotted for signal intensity for each pixel (mass spectrum) across a given area (tissue section) using MSiReader software.^86^ The *m*/*z* range of interest was normalized using the ratio of the total ion current for each mass spectrum. The intensity reached by each peak of the spectrum (*m*/*z* or molecule) was further calculated as a ratio of the peak with the highest intensity, and the average was obtained using OriginPro8 (Northampton, MA, USA) software. The most intense peak (*m*/*z* or molecule) was considered to be 100%. In the organotypic cultures the most intense peak was modified after treatments; therefore the data were expressed as absolute intensity in arbitrary units.

The assignment of lipid species was facilitated by the use of the databases Lipid MAPS (http://www.lipidmaps.org/) and the Human Metabolome Database (HMDB) (https://hmdb.ca). 5 ppm mass accuracy was selected as the tolerance window for the assignment.

### Statistical Analyses

Step-through latencies were represented as Kaplan–Meier survival curves, and for comparisons between groups, the log-rank/Mantel–Cox test was used. Kolmogorov–Smirnov analyses were run to check the Gaussian distribution of the data obtained. Acquisition latencies and absolute intensity of lipids were analyzed by one-way analysis of variance (ANOVA) followed by Bonferroni′s post hoc test for multiple comparisons and Spearman rank for correlations (Sigma Plot 12.5, Systat Software, San Jose, CA). AChE densities, the percentages of agonist-evoked [^35^S]GTPγS stimulation, the density of M_1_ and M_2_ mAChR (fmol/mg t.e.), the electric-shock-evoked first vocalization, and the percentages of the relative intensity of lipids were statistically analyzed using the two-tailed unpaired Student *t* test. The statistical analyses were performed by using the GraphPad Prism 5.01 software. The statistical significance threshold was set at *p* < 0.05.

## Supporting Information Available

Table S1 listing relative intensity of representative lipid species that have been found to be modified in the NBM and cortex of vehicle (aCSF) and 192IgG-saporin treated rats; Tables S2, S3, and S4 listing [^35^S]GTPγS basal and carbachol-induced binding in different brain areas; Table S5 listing [^3^H]-pirenzepine and [^3^H]-oxotremorine binding in different brain regions; Table S6 listing absolute intensity of lipid species in organotypic cultures (PDF)

## Abbreviations

AA: arachidonic acid
ACh: acetylcholine
AChE: acetylcholinesterase
aCSF: artificial cerebrospinal fluid
AD: Alzheimer’s disease
BFCN: basal forebrain cholinergic neurons
Ch: choline
ChAT: choline acetyltransferase
DAG: diacylglycerol
DHA: docosahexaenoic acid
GTPγS: guanosine 5′-*O*-3-thiotriphosphate
mAChR: muscarinic acetylcholine receptor
M_1_ mAChR: subtype-1 of muscarinic acetylcholine receptor
M_2_ mAChR: subtype-2 of muscarinic acetylcholine receptor
M_4_ mAChR: subtype-4 of muscarinic acetylcholine receptor
MALDI-IMS: matrix-assisted laser desorption ionization imaging mass spectrometry
NBM: nucleus basalis magnocellularis
PA: phosphatidic acid
PC: phosphatidylcholine
PL: phospholipid
PLA2: phospholipase A2
LPC: lysophosphatidylcholine
PE: phosphatidylethanolamine
PG: phosphoglycerol
PS: phosphatidylserine
PUFA: polyunsaturated fatty acid
RRID: research resource identifier (see scicrunch.org)
SAP: 192IgG-saporin treated rat
SM: sphingomyelin

## References

[ref1] Davies P.; Maloney A. J. (1976) Selective Loss of Central Cholinergic Neurons in Alzheimer’s Disease. Lancet 308, 1403 10.1016/S0140-6736(76)91936-X. 63862

[ref2] Perry E. K.; Gibson P. H.; Blessed G.; Perry R. H.; Tomlinson B. E. (1977) Neurotransmitter Enzyme Abnormalities in Senile Dementia. Choline Acetyltransferase and Glutamic Acid Decarboxylase Activities in Necropsy Brain Tissue. J. Neurol. Sci. 34 (2), 247–265. 10.1016/0022-510X(77)90073-9. 144789

[ref3] Whitehouse P. J.; Price D. L.; Struble R. G.; Clark A. W.; Coyle J. T.; Delon M. R. (1982) Alzheimer’s Disease and Senile Dementia: Loss of Neurons in the Basal Forebrain. Science 215 (4537), 1237–1239. 10.1126/science.7058341. 7058341

[ref4] Rodríguez-Puertas R.; Pazos A.; Zarranz J. J.; Pascual J. (1994) Selective Cortical Decrease of High-Affinity Choline Uptake Carrier in Alzheimer’s Disease: An Autoradiographic Study Using 3H-Hemicholinium-3. J. Neural Transm.: Parkinson's Dis. Dementia Sect. 8 (3), 161–169. 10.1007/BF02260937. 7748460

[ref5] Rodríguez-Puertas R.; Pascual J.; Vilaró T.; Pazos A. (1997) Autoradiographic Distribution of M1, M2, M3, and M4Muscarinic Receptor Subtypes in Alzheimer’s Disease. Synapse 26 (4), 341–350. 10.1002/(SICI)1098-2396(199708)26:4<341::AID-SYN2>3.0.CO;2-6. 9215593

[ref6] Avery E. E.; Baker L. D.; Asthana S. (1997) Potential Role of Muscarinic Agonists in Alzheimer’s Disease. Drugs Aging 11 (6), 450–459. 10.2165/00002512-199711060-00004. 9413702

[ref7] Drachman D. A.; Leavitt J. (1974) Human Memory and the Cholinergic System. A Relationship to Aging?. Arch. Neurol. 30 (2), 113–121. 10.1001/archneur.1974.00490320001001. 4359364

[ref8] Bartus R. T.; Dean R. L. 3rd; Beer B.; Lippa A. S. (1982) The Cholinergic Hypothesis of Geriatric Memory Dysfunction. Science 217 (4558), 408–414. 10.1126/science.7046051. 7046051

[ref9] Millington W. R.; Wurtman R. J. (1982) Choline Administration Elevates Brain Phosphorylcholine Concentrations. J. Neurochem. 38 (6), 1748–1752. 10.1111/j.1471-4159.1982.tb06658.x. 7077335

[ref10] Ulus I. H.; Wurtman R. J.; Mauron C.; Blusztajn J. K. (1989) Choline Increases Acetylcholine Release and Protects against the Stimulation-Induced Decrease in Phosphatide Levels within Membranes of Rat Corpus Striatum. Brain Res. 484 (1–2), 217–227. 10.1016/0006-8993(89)90364-8. 2713682

[ref11] Wurtman R. J.; Blusztajn J. K.; Maire J. C. (1985) “Autocannibalism” of Choline-Containing Membrane Phospholipids in the Pathogenesis of Alzheimer’s Disease-A Hypothesis. Neurochem. Int. 7 (2), 369–372. 10.1016/0197-0186(85)90127-5. 20492936

[ref12] Qian Z.; Drewes L. R. (1989) Muscarinic Acetylcholine Receptor Regulates Phosphatidylcholine Phospholipase D in Canine Brain. J. Biol. Chem. 264 (36), 21720–21724. 10.1016/S0021-9258(20)88245-3. 2513326

[ref13] Dolezal V.; Tucek S. (1984) Activation of Muscarinic Receptors Stimulates the Release of Choline from Brain Slices. Biochem. Biophys. Res. Commun. 120 (3), 1002–1007. 10.1016/S0006-291X(84)80206-5. 6732780

[ref14] Maire J. C.; Wurtman R. J. (1985) Effects of Electrical Stimulation and Choline Availability on the Release and Contents of Acetylcholine and Choline in Superfused Slices from Rat Striatum. J. Physiol. (Paris) 80 (3), 189–195. 3003350

[ref15] Wiley R. G.; Oeltmann T. N.; Lappi D. A. (1991) Immunolesioning: Selective Destruction of Neurons Using Immunotoxin to Rat NGF Receptor. Brain Res. 562 (1), 149–153. 10.1016/0006-8993(91)91199-B. 1666014

[ref16] Rossner S. (1997) Cholinergic Immunolesions by 192IgG-Saporin--Useful Tool to Simulate Pathogenic Aspects of Alzheimer’s Disease. Int. J. Dev. Neurosci. 15 (7), 835–850. 10.1016/S0736-5748(97)00035-X. 9568532

[ref17] Llorente-Ovejero A.; Manuel I.; Giralt M. T.; Rodríguez-Puertas R. (2017) Increase in Cortical Endocannabinoid Signaling in a Rat Model of Basal Forebrain Cholinergic Dysfunction. Neuroscience 362, 206–218. 10.1016/j.neuroscience.2017.08.008. 28827178

[ref18] Baxter M. G. (2000) Effects of Selective Immunotoxic Lesions on Learning and Memory. Methods Mol. Biol. 166, 249–265. 10.1385/1-59259-114-0:249. 11217371

[ref19] Berger-Sweeney J.; Heckers S.; Mesulam M. M.; Wiley R. G.; Lappi D. A.; Sharma M. (1994) Differential Effects on Spatial Navigation of Immunotoxin-Induced Cholinergic Lesions of the Medial Septal Area and Nucleus Basalis Magnocellularis. J. Neurosci. 14 (7), 4507–4519. 10.1523/JNEUROSCI.14-07-04507.1994. 8027790 PMC6577026

[ref20] Moreno-Rodríguez M., Martínez-Gardeazabal J., Llorente-Ovejero A., Lombardero L., Manuel I., and Rodríguez-Puertas R. (2018) Learning and Memory Improvement Mediated by CB1 Cannabinoid Receptors in Animal Models of Cholinergic Dysfunction. Abstracts, Society for Neuroscience (SFN) Symposium, San Diego, November 3–7, 2018, Program No. 049.05/S3, Society for Neuroscience.

[ref21] Pettegrew J. W.; Panchalingam K.; Hamilton R. L.; McClure R. J. (2001) Brain Membrane Phospholipid Alterations in Alzheimer’s Disease. Neurochem. Res. 26 (7), 771–782. 10.1023/A:1011603916962. 11565608

[ref22] Martín V.; Fabelo N.; Santpere G.; Puig B.; Marín R.; Ferrer I.; Díaz M. (2010) Lipid Alterations in Lipid Rafts from Alzheimer’s Disease Human Brain Cortex. J. Alzheimer's Dis. 19 (2), 489–502. 10.3233/JAD-2010-1242. 20110596

[ref23] Mapstone M.; Cheema A. K.; Fiandaca M. S.; Zhong X.; Mhyre T. R.; MacArthur L. H.; Hall W. J.; Fisher S. G.; Peterson D. R.; Haley J. M.; et al. (2014) Plasma Phospholipids Identify Antecedent Memory Impairment in Older Adults. Nat. Med. 20 (4), 415–418. 10.1038/nm.3466. 24608097 PMC5360460

[ref24] Chatterjee P.; Lim W. L. F.; Shui G.; Gupta V. B.; James I.; Fagan A. M.; Xiong C.; Sohrabi H. R.; Taddei K.; Brown B. M.; et al. (2016) Plasma Phospholipid and Sphingolipid Alterations in Presenilin1 Mutation Carriers: A Pilot Study. J. Alzheimer's Dis. 50 (3), 887–894. 10.3233/JAD-150948. 26836186 PMC4943576

[ref25] Fiandaca M. S.; Zhong X.; Cheema A. K.; Orquiza M. H.; Chidambaram S.; Tan M. T.; Gresenz C. R.; FitzGerald K. T.; Nalls M. A.; Singleton A. B.; et al. (2015) Plasma 24-Metabolite Panel Predicts Preclinical Transition to Clinical Stages of Alzheimer’s Disease. Front. Neurol. 6, 237 10.3389/fneur.2015.00237. 26617567 PMC4642213

[ref26] Dorninger F.; Moser A. B.; Kou J.; Wiesinger C.; Forss-Petter S.; Gleiss A.; Hinterberger M.; Jungwirth S.; Fischer P.; Berger J. (2018) Alterations in the Plasma Levels of Specific Choline Phospholipids in Alzheimer’s Disease Mimic Accelerated Aging. J. Alzheimer's Dis. 62 (2), 841–854. 10.3233/JAD-171036. 29480199 PMC5837024

[ref27] Gónzalez de San Román E.; Manuel I.; Giralt M. T.; Ferrer I.; Rodríguez-Puertas R. (2017) Imaging Mass Spectrometry (IMS) of Cortical Lipids from Preclinical to Severe Stages of Alzheimer’s Disease. Biochim. Biophys. Acta, Biomembr. 1859 (9, Part B), 1604–1614. 10.1016/j.bbamem.2017.05.009. 28527668

[ref28] Amenta F.; Parnetti L.; Gallai V.; Wallin A. (2001) Treatment of Cognitive Dysfunction Associated with Alzheimer’s Disease with Cholinergic Precursors. Ineffective Treatments or Inappropriate Approaches?. Mech. Ageing Dev. 122 (16), 2025–2040. 10.1016/S0047-6374(01)00310-4. 11589920

[ref29] Hampel H.; Mesulam M.-M.; Cuello A. C.; Farlow M. R.; Giacobini E.; Grossberg G. T.; Khachaturian A. S.; Vergallo A.; Cavedo E.; Snyder P. J.; et al. (2018) The Cholinergic System in the Pathophysiology and Treatment of Alzheimer’s Disease. Brain 141 (7), 1917–1933. 10.1093/brain/awy132. 29850777 PMC6022632

[ref30] Martínez-Gardeazabal J.; González de San Román E.; Moreno-Rodríguez M.; Llorente-Ovejero A.; Manuel I.; Rodríguez-Puertas R. (2017) Lipid Mapping of the Rat Brain for Models of Disease. Biochim. Biophys. Acta, Biomembr. 1859 (9, Part B), 1548–1557. 10.1016/j.bbamem.2017.02.011. 28235468

[ref31] Hino K.; Kaneko S.; Harasawa T.; Kimura T.; Takei S.; Shinohara M.; Yamazaki F.; Morita S.-Y.; Sato S.; Kubo Y.; et al. (2019) Change in Brain Plasmalogen Composition by Exposure to Prenatal Undernutrition Leads to Behavioral Impairment of Rats. J. Neurosci. 39 (39), 7689–7702. 10.1523/JNEUROSCI.2721-18.2019. 31391260 PMC6764206

[ref32] Vierck C. J.; Yezierski R. P.; Wiley R. G. (2016) Pain Sensitivity Following Loss of Cholinergic Basal Forebrain (CBF) Neurons in the Rat. Neuroscience 319, 23–34. 10.1016/j.neuroscience.2016.01.038. 26812034

[ref33] Barreda-Gómez G.; Lombardero L.; Giralt M. T.; Manuel I.; Rodríguez-Puertas R. (2015) Effects of Galanin Subchronic Treatment on Memory and Muscarinic Receptors. Neuroscience 293, 23–34. 10.1016/j.neuroscience.2015.02.039. 25732139

[ref34] Torres E. M.; Perry T. A.; Blokland A.; Wilkinson L. S.; Wiley R. G.; Lappi D. A.; Dunnett S. B. (1994) Behavioural, Histochemical and Biochemical Consequences of Selective Immunolesions in Discrete Regions of the Basal Forebrain Cholinergic System. Neuroscience 63 (1), 95–122. 10.1016/0306-4522(94)90010-8. 7898665

[ref35] Zhang Z. J.; Berbos T. G.; Wrenn C. C.; Wiley R. G. (1996) Loss of Nucleus Basalis Magnocellularis, but Not Septal, Cholinergic Neurons Correlates with Passive Avoidance Impairment in Rats Treated with 192-Saporin. Neurosci. Lett. 203 (3), 214–218. 10.1016/0304-3940(95)12282-6. 8742031

[ref36] Wenk G. L.; Stoehr J. D.; Quintana G.; Mobley S.; Wiley R. G. (1994) Behavioral, Biochemical, Histological, and Electrophysiological Effects of 192 IgG-Saporin Injections into the Basal Forebrain of Rats. J. Neurosci. 14 (10), 5986–5995. 10.1523/JNEUROSCI.14-10-05986.1994. 7523630 PMC6576971

[ref37] Jackson S. N.; Wang H.-Y. J.; Woods A. S.; Ugarov M.; Egan T.; Schultz J. A. (2005) Direct Tissue Analysis of Phospholipids in Rat Brain Using MALDI-TOFMS and MALDI-Ion Mobility-TOFMS. J. Am. Soc. Mass Spectrom. 16 (2), 133–138. 10.1016/j.jasms.2004.10.002. 15694763

[ref38] Astigarraga E.; Barreda-Gómez G.; Lombardero L.; Fresnedo O.; Castaño F.; Giralt M. T.; Ochoa B.; Rodríguez-Puertas R.; Fernández J. A. (2008) Profiling and Imaging of Lipids on Brain and Liver Tissue by Matrix-Assisted Laser Desorption/ Ionization Mass Spectrometry Using 2-Mercaptobenzothiazole as a Matrix. Anal. Chem. 80 (23), 9105–9114. 10.1021/ac801662n. 18959430

[ref39] Veloso A.; Astigarraga E.; Barreda-Gómez G.; Manuel I.; Ferrer I.; Giralt M. T.; Ochoa B.; Fresnedo O.; Rodríguez-Puertas R.; Fernández J. A. (2011) Anatomical Distribution of Lipids in Human Brain Cortex by Imaging Mass Spectrometry. J. Am. Soc. Mass Spectrom. 22 (2), 329–338. 10.1007/s13361-010-0024-5. 21472592

[ref40] Minami M.; Kimura S.; Endo T.; Hamaue N.; Hirafuji M.; Togashi H.; Matsumoto M.; Yoshioka M.; Saito H.; Watanabe S.; et al. (1997) Dietary Docosahexaenoic Acid Increases Cerebral Acetylcholine Levels and Improves Passive Avoidance Performance in Stroke-Prone Spontaneously Hypertensive Rats. Pharmacol., Biochem. Behav. 58 (4), 1123–1129. 10.1016/S0091-3057(97)00300-6. 9408223

[ref41] Almeida T.; Cunha R. A.; Ribeiro J. A. (1999) Facilitation by Arachidonic Acid of Acetylcholine Release from the Rat Hippocampus. Brain Res. 826 (1), 104–111. 10.1016/S0006-8993(99)01267-6. 10216201

[ref42] Wurtman R. J.; Ulus I. H.; Cansev M.; Watkins C. J.; Wang L.; Marzloff G. (2006) Synaptic Proteins and Phospholipids Are Increased in Gerbil Brain by Administering Uridine plus Docosahexaenoic Acid Orally. Brain Res. 1088 (1), 83–92. 10.1016/j.brainres.2006.03.019. 16631143

[ref43] Calzada E.; Onguka O.; Claypool S. M. (2016) Phosphatidylethanolamine Metabolism in Health and Disease. Int. Rev. Cell Mol. Biol. 321, 29–88. 10.1016/bs.ircmb.2015.10.001. 26811286 PMC4778737

[ref44] Bremer J.; Figard P. H.; Greenberg D. M. (1960) The Biosynthesis of Choline and Its Relation to Phospholipid Metabolism. Biochim. Biophys. Acta 43 (C), 477–488. 10.1016/0006-3002(60)90470-4.

[ref45] Hattori H.; Kanfer J. N. (1985) Synaptosomal Phospholipase D Potential Role in Providing Choline for Acetylcholine Synthesis. J. Neurochem. 45 (5), 1578–1584. 10.1111/j.1471-4159.1985.tb07229.x. 4045465

[ref46] Blusztajn J. K.; Liscovitch M.; Richardson U. I. (1987) Synthesis of Acetylcholine from Choline Derived from Phosphatidylcholine in a Human Neuronal Cell Line. Proc. Natl. Acad. Sci. U. S. A. 84 (15), 5474–5477. 10.1073/pnas.84.15.5474. 3474663 PMC298880

[ref47] Exton J. H. (1999) Regulation of Phospholipase D. Biochim. Biophys. Acta, Mol. Cell Biol. Lipids 1439 (2), 121–133. 10.1016/S1388-1981(99)00089-X. 10425390

[ref48] Kanfer J. N.; Hattori H.; Orihel D. (1986) Reduced Phospholipase D Activity in Brain Tissue Samples from Alzheimer’s Disease Patients. Ann. Neurol. 20 (2), 265–267. 10.1002/ana.410200214. 3019230

[ref49] Fadok V. A.; Voelker D. R.; Campbell P. A.; Cohen J. J.; Bratton D. L.; Henson P. M. (1992) Exposure of Phosphatidylserine on the Surface of Apoptotic Lymphocytes Triggers Specific Recognition and Removal by Macrophages. J. Immunol. 148 (7), 2207–2216. 1545126

[ref50] Bader Lange M. L.; Cenini G.; Piroddi M.; Abdul H. M.; Sultana R.; Galli F.; Memo M.; Butterfield D. A. (2008) Loss of Phospholipid Asymmetry and Elevated Brain Apoptotic Protein Levels in Subjects with Amnestic Mild Cognitive Impairment and Alzheimer Disease. Neurobiol. Dis. 29 (3), 456–464. 10.1016/j.nbd.2007.11.004. 18077176 PMC2292396

[ref51] Bevers E. M.; Williamson P. L. (2016) Getting to the Outer Leaflet: Physiology of Phosphatidylserine Exposure at the Plasma Membrane. Physiol. Rev. 96 (2), 605–645. 10.1152/physrev.00020.2015. 26936867

[ref52] Wymann M. P.; Schneiter R. (2008) Lipid Signalling in Disease. Nat. Rev. Mol. Cell Biol. 9 (2), 162–176. 10.1038/nrm2335. 18216772

[ref53] Han X.; Rozen S.; Boyle S. H.; Hellegers C.; Cheng H.; Burke J. R.; Welsh-Bohmer K. A.; Doraiswamy P. M.; Kaddurah-Daouk R. (2011) Metabolomics in Early Alzheimer’s Disease: Identification of Altered Plasma Sphingolipidome Using Shotgun Lipidomics. PLoS One 6 (7), e21643 10.1371/journal.pone.0021643. 21779331 PMC3136924

[ref54] Fonteh A. N.; Ormseth C.; Chiang J.; Cipolla M.; Arakaki X.; Harrington M. G. (2015) Sphingolipid Metabolism Correlates with Cerebrospinal Fluid Beta Amyloid Levels in Alzheimer’s Disease. PLoS One 10 (5), e0125597 10.1371/journal.pone.0125597. 25938590 PMC4418746

[ref55] Koal T.; Klavins K.; Seppi D.; Kemmler G.; Humpel C. (2015) Sphingomyelin SM(D18:1/18:0) Is Significantly Enhanced in Cerebrospinal Fluid Samples Dichotomized by Pathological Amyloid-B42, Tau, and Phospho-Tau-181 Levels. J. Alzheimer's Dis. 44 (4), 1193–1201. 10.3233/JAD-142319. 25408209 PMC4699259

[ref56] Mendis L. H. S.; Grey A. C.; Faull R. L. M.; Curtis M. A. (2016) Hippocampal Lipid Differences in Alzheimer’s Disease: A Human Brain Study Using Matrix-Assisted Laser Desorption/Ionization-Imaging Mass Spectrometry. Brain Behav. 6 (10), e00517 10.1002/brb3.517. 27781133 PMC5064331

[ref57] Mash D. C.; Flynn D. D.; Potter L. T. (1985) Loss of M2 Muscarine Receptors in the Cerebral Cortex in Alzheimer’s Disease and Experimental Cholinergic Denervation. Science 228 (4703), 1115–1117. 10.1126/science.3992249. 3992249

[ref58] Rossner S.; Schliebs R.; Perez-Polo J. R.; Wiley R. G.; Bigl V. (1995) Differential Changes in Cholinergic Markers from Selected Brain Regions after Specific Immunolesion of the Rat Cholinergic Basal Forebrain System. J. Neurosci. Res. 40 (1), 31–43. 10.1002/jnr.490400105. 7714924

[ref59] Mesulam M. M. (1998) Some Cholinergic Themes Related to Alzheimer’s Disease: Synaptology of the Nucleus Basalis, Location of M2 Receptors, Interactions with Amyloid Metabolism, and Perturbations of Cortical Plasticity. J. Physiol. 92 (3–4), 293–298. 10.1016/S0928-4257(98)80036-3. 9789826

[ref60] Mrzljak L.; Levey A. I.; Belcher S.; Goldman-Rakic P. S. (1998) Localization of the M2 Muscarinic Acetylcholine Receptor Protein and MRNA in Cortical Neurons of the Normal and Cholinergically Deafferented Rhesus Monkey. J. Comp. Neurol. 390 (1), 112–132. 10.1002/(SICI)1096-9861(19980105)390:1<112::AID-CNE10>3.0.CO;2-Z. 9456180

[ref61] Raevsky V. V.; Dawe G. S.; Sinden J. D.; Stephenson J. D. (1998) Lesions of the Nucleus Basalis Magnocellularis Do Not Alter the Proportions of Pirenzepine- and Gallamine-Sensitive Responses of Somatosensory Cortical Neurones to Acetylcholine in the Rat. Brain Res. 782 (1–2), 324–328. 10.1016/S0006-8993(97)01364-4. 9519281

[ref62] Bauer A.; Schulz J. B.; Zilles K. (1992) Muscarinic Desensitization after Septal Lesions in Rat Hippocampus: Evidence for the Involvement of G-Proteins. Neuroscience 47 (1), 95–103. 10.1016/0306-4522(92)90124-K. 1579209

[ref63] Potter P. E.; Gaughan C.; Assouline Y. (1999) Lesion of Septal-Hippocampal Neurons with 192 IgG-Saporin Alters Function of M1Muscarinic Receptors. Neuropharmacology 38 (4), 579–586. 10.1016/S0028-3908(98)00207-X. 10221761

[ref64] Wall S. J.; Wolfe B. B.; Kromer L. F. (1994) Cholinergic Deafferentation of Dorsal Hippocampus by Fimbria-Fornix Lesioning Differentially Regulates Subtypes (M1-M5) of Muscarinic Receptors. J. Neurochem. 62 (4), 1345–1351. 10.1046/j.1471-4159.1994.62041345.x. 8133265

[ref65] Levey A. I.; Edmunds S. M.; Koliatsos V.; Wiley R. G.; Heilman C. J. (1995) Expression of M1-M4 Muscarinic Acetylcholine Receptor Proteins in Rat Hippocampus and Regulation by Cholinergic Innervation. J. Neurosci. 15 (5), 4077–4092. 10.1523/JNEUROSCI.15-05-04077.1995. 7751967 PMC6578239

[ref66] Köppen J. R.; Stuebing S. L.; Sieg M. L.; Blackwell A. A.; Blankenship P. A.; Cheatwood J. L.; Wallace D. G. (2016) Cholinergic Deafferentation of the Hippocampus Causes Non-Temporally Graded Retrograde Amnesia in an Odor Discrimination Task. Behav. Brain Res. 299, 97–104. 10.1016/j.bbr.2015.11.021. 26611564

[ref67] Raiteri M.; Leardi R.; Marchi M. (1984) Heterogeneity of Presynaptic Muscarinic Receptors Regulating Neurotransmitter Release in the Rat Brain. J. Pharmacol. Exp. Ther. 228 (1), 209–214. 6141277

[ref68] Hájos N.; Papp E. C.; Acsády L.; Levey A. I.; Freund T. F. (1997) Distinct Interneuron Types Express M2 Muscarinic Receptor Immunoreactivity on Their Dendrites or Axon Terminals in the Hippocampus. Neuroscience 82 (2), 355–376. 10.1016/S0306-4522(97)00300-X. 9466448

[ref69] Zhao L.-X.; Ge Y.-H.; Xiong C.-H.; Tang L.; Yan Y.-H.; Law P.-Y.; Qiu Y.; Chen H.-Z. (2018) M1 Muscarinic Receptor Facilitates Cognitive Function by Interplay with AMPA Receptor GluA1 Subunit. FASEB J. 32 (8), 4247–4257. 10.1096/fj.201800029R. 29509512

[ref70] Tzavara E. T.; Bymaster F. P.; Felder C. C.; Wade M.; Gomeza J.; Wess J.; McKinzie D. L.; Nomikos G. G. (2003) Dysregulated Hippocampal Acetylcholine Neurotransmission and Impaired Cognition in M2, M4 and M2/M4 Muscarinic Receptor Knockout Mice. Mol. Psychiatry 8 (7), 673–679. 10.1038/sj.mp.4001270. 12874603

[ref71] Seeger T.; Fedorova I.; Zheng F.; Miyakawa T.; Koustova E.; Gomeza J.; Basile A. S.; Alzheimer C.; Wess J. (2004) M2 Muscarinic Acetylcholine Receptor Knock-out Mice Show Deficits in Behavioral Flexibility, Working Memory, and Hippocampal Plasticity. J. Neurosci. 24 (45), 10117–10127. 10.1523/JNEUROSCI.3581-04.2004. 15537882 PMC6730182

[ref72] Segal M.; Auerbach J. M. (1997) Muscarinic Receptors Involved in Hippocampal Plasticity. Life Sci. 60 (13–14), 1085–1091. 10.1016/S0024-3205(97)00051-9. 9121351

[ref73] Peng X.; Frohman M. A. (2012) Mammalian Phospholipase D Physiological and Pathological Roles. Acta Physiol. 204 (2), 219–226. 10.1111/j.1748-1716.2011.02298.x. PMC3137737 21447092

[ref74] Burkhardt U.; Stegner D.; Hattingen E.; Beyer S.; Nieswandt B.; Klein J. (2014) Impaired Brain Development and Reduced Cognitive Function in Phospholipase D-Deficient Mice. Neurosci. Lett. 572, 48–52. 10.1016/j.neulet.2014.04.052. 24813107

[ref75] Bunney T. D.; Katan M. (2011) PLC Regulation: Emerging Pictures for Molecular Mechanisms. Trends Biochem. Sci. 36 (2), 88–96. 10.1016/j.tibs.2010.08.003. 20870410

[ref76] Rebecchi M. J.; Pentyala S. N. (2000) Structure, Function, and Control of Phosphoinositide-Specific Phospholipase C. Physiol. Rev. 80 (4), 1291–1335. 10.1152/physrev.2000.80.4.1291. 11015615

[ref77] Reese J. H.; Hoss W. (1983) Activation of Fluoride-Stimulated Adenylate Cyclase by Phospholipase A2 in the Caudate Nucleus of the Rat Brain. Neurochem. Res. 8 (8), 1059–1069. 10.1007/BF00965200. 6621779

[ref78] Farooqui A. A.; Horrocks L. A. (2004) Brain Phospholipases A2: A Perspective on the History. Prostaglandins, Leukotrienes Essent. Fatty Acids 71 (3), 161–169. 10.1016/j.plefa.2004.03.004. 15253885

[ref79] Bayon Y.; Hernandez M.; Alonso A.; Nuñez L.; Garcia-Sancho J.; Leslie C.; Sanchez Crespo M.; Nieto M. L. (1997) Cytosolic Phospholipase A2 Is Coupled to Muscarinic Receptors in the Human Astrocytoma Cell Line 1321N1: Characterization of the Transducing Mechanism. Biochem. J. 323 (1), 281–287. 10.1042/bj3230281. 9173894 PMC1218307

[ref80] Blusztajn J. K.; Liscovitch M.; Mauron C.; Richardson U. I.; Wurtman R. J. (1987) Phosphatidylcholine as a Precursor of Choline for Acetylcholine Synthesis. J. Neural Transm. Suppl. 24, 247–259. 3316498

[ref81] Stoppini L.; Buchs P. A.; Muller D. (1991) A Simple Method for Organotypic Cultures of Nervous Tissue. J. Neurosci. Methods 37 (2), 173–182. 10.1016/0165-0270(91)90128-M. 1715499

[ref82] Paxinos G., and Watson C. (2007) The Rat Brain in Stereotaxic Coordinates, 6th ed., Academic Press, New York.

[ref83] Karnovsky M. J.; Roots L. (1964) A “Direct-Coloring” Thiocholine Method for Cholinesterases. J. Histochem. Cytochem. 12, 219–221. 10.1177/12.3.219. 14187330

[ref84] Schwartz S. A.; Reyzer M. L.; Caprioli R. M. (2003) Direct Tissue Analysis Using Matrix-Assisted Laser Desorption/Ionization Mass Spectrometry: Practical Aspects of Sample Preparation. J. Mass Spectrom. 38 (7), 699–708. 10.1002/jms.505. 12898649

[ref85] Bradford M. M. (1976) A Rapid and Sensitive Method for the Quantitation of Microgram Quantities of Protein Utilizing the Principle of Protein-Dye Binding. Anal. Biochem. 72, 248–254. 10.1006/abio.1976.9999. 942051

[ref86] Robichaud G.; Garrard K. P.; Barry J. A.; Muddiman D. C. (2013) MSiReader: An Open-Source Interface to View and Analyze High Resolving Power MS Imaging Files on Matlab Platform. J. Am. Soc. Mass Spectrom. 24 (5), 718–721. 10.1007/s13361-013-0607-z. 23536269 PMC3693088

