# Supplementary material for: Specific Phospholipid Modulation by Muscarinic Signaling in a Rat Lesion Model of Alzheimer’s Disease
Source: ACS Chem Neurosci. 2021 May 26;12(12):2167–81. doi: 10.1021/acschemneuro.1c00169 (PMC9162383; doi:10.1021/acschemneuro.1c00169)
Supplement: Supplementary file 1 — cn1c00169_si_001.pdf [file cn1c00169_si_001.pdf]

Supporting information

Specific Phospholipid Modulation by Muscarinic Signaling in a Rat Lesion Model of Alzheimer's Disease

Alberto Llorente-Ovejero[1], Jonatan Martínez-Gardeazabal[1], Marta Moreno-Rodríguez[1], Laura Lombardero[1], Estíbaliz González de San Román[1], Iván Manuel[1,2], María Teresa Giralt[1], Rafael Rodríguez-Puertas[1,2] *

[1]Department of Pharmacology, Faculty of Medicine and Nursing. University of the Basque Country (UPV/EHU), B° Sarriena s/n, 48940 Leioa, Spain.

[2]Neurodegenerative Diseases, BioCruces Bizkaia Health Research Institute, Barakaldo, Spain.

Table S1. Relative intensity of representative lipid species which have been found to be modified in the NBM and cortex of vehicle (aCSF) and 192IgG-saporin treated rats, in both positive and negative ion mode.

			% Intensity[1]			
			NBM		Cortex	
**Assignment**	**Cal m/z**	**Exp m/z**	aCSF	**SAP**	aCSF	**SAP**
**Positive ion mode**						
PE (14:1/20:4) + H^+	710.4755	710.4893	15.21 ± 0.70	**11.87 ± 1.38***	9.97 ± 0.62	**10.67 ± 0.67**
SM (d18:1/18:0) + H^+	731.6061	731.6070	7.11 ± 1.13	**4.15 ± 0.80***	4.19 ± 0.44	**3.50 ± 0.53**
SM (d18:1/16:0) + K^+	741.5313	741.5311	0.96 ± 0.14	**10.63 ± 3.33****	0.55 ± 0.13	**0.78 ± 0.11**
PC (32:0)+Na^+/PC (34:3) + H^+	756.5514	756.5520	15.75 ± 1.23	**21.94 ± 2.46***	16.94 ± 1.02	**17.41 ± 1.31**
PC (34:2)+Na^+/PC (36:5) + H^+	780.5514	780.5521	0.85 ± 0.09	**2.02 ± 0.27****	1.10 ± 0.08	**1.07 ± 0.08**
PC (34:1)+Na^+/PC (36:4) + H^+	782.5670	782.5673	32.75 ± 1.65	**46.10 ± 3.86****	32.31 ± 1.64	**30.9 ± 1.29**
PC (34:2) + K^+	796.5253	796.5255	3.27 ± 0.17	**5.2 ± 0.30*****	4.20 ± 0.15	**4.16 ± 0.07**
PC (34:1) + K^+	798.5410	798.5405	100 ± 0	**100 ± 0**	100 ± 0	**100 ± 0**
PC (36:4)+Na^+/PC (38:7) + H^+	804.5514	804.5510	2.79 ± 0.31	**7.69 ± 1.52***	3.63 ± 0.31	**4.3 ± 0.24**
PC (36:1)+Na^+/PC (38:4) + H^+	810.5983	810.5984	14.62 ± 0.30	**21.64 ± 1.53****	10.62 ± 0.48	**8.95 ± 0.63***
PC (36:4) + K^+	820.5253	820.5249	10.32 ± 1.01	**18.61 ± 2.71***	13.17 ± 0.99	**15.63 ± 0.48***
PC (38:4)+Na^+/PC (40:7) + H^+	832.5827	832.5826	3.00 ± 0.30	**7.98 ± 1.40****	2.82 ± 0.25	**3.11 ± 0.21**
PC (38:4)+K^+/PC (42:9) + H^+	848.5566	848.5560	10.23 ± 0.93	**18.27 ± 2.15****	9.71 ± 0.80	**10.75 ± 0.38**
PC (40:6) + Na^+	856.5827	856.5820	0.88 ± 0.17	**2.25 ± 0.40****	0.75 ± 0.11	**1.14 ± 0.09***
**Negative ion mode**						
SM (d18:1/15:0) - H^-	687.5447	687.5443	1.26 ± 0.24	**6.44 ± 1.50****	0.94 ± 0.23	**1.52 ± 0.28**
PS (18:0/18:1) - H^-	788.5447	788.5447	11.04 ± 2.16	**3.95 ± 0.99****	8.54 ± 1.28	**6.30 ± 0.85**
PE (40:4)/PC (18:0/20:4) - H^-	794.5705	794.5705	0.82 ± 0.13	**1.35 ± 0.17***	0.83 ± 0.08	**0.98 ± 0.14**
PG (22:6/22:6) - H^-	865.5025	865.5033	0.06 ± 0.01	**0.86 ± 0.24****	0.04 ± 0.01	**0.16 ± 0.05***

[1] The maximal peak is the most intense peak of the lipid spectrum, in this case PC (34:1) in positive and PI (20:4/18:0) in negative ion modes, which are set at 100%. Data are mean ± S.E.M values of aCSF (n = 8) and 192IgG-saporin-treated (n = 8) rats. *p < 0.05, **p < 0.01 and ***p < 0.001 when compared to aCSF group. PC: phosphatidylcholine; SM: sphingomyelin; PS: phosphatidylserine; PE: phosphatidylethanolamine; PG: phosphoglycerol; PI: phosphatidylinositol; Cal: calculated; Exp: experimental.

Table S2. [^{35}S]GTPγS basal and carbachol-induced (100 µM) binding in cortical, hippocampal and several brain regions of vehicle (aCSF) and 192IgG-saporin-treated rats.

	Basal binding (nCi/g t.e.)		**Carbachol stimulation (% Over basal)**	
**Brain region**	aCSF	SAP	**aCSF**	**SAP**
**Cerebral cortex**				
Cingulate	304 ± 26	306 ± 20	**52 ± 12.1**	**48 ± 6.7**
Ectorhinal	352 ± 34	290 ± 29	**66 ± 12.5**	**58 ± 8.0**
Entorhinal	342 ± 35	335 ± 36	**76 ± 10.5**	**93 ± 11.6**
Perirhinal	338 ± 49	309 ± 29	**35 ± 5.8**	**43 ± 3.5**
Somatosensory	355 ± 28	333 ± 20	**31 ± 7.0**	**54 ± 10.6**
Motor	311 ± 33	319 ± 20	**40 ± 13.3**	**66 ± 6.5**
**Hippocampus**				
CA1				
Oriens	320 ± 40	269 ± 32	**26 ± 9.4**	**40 ± 10.4**
Pyramidal	527 ± 64	457 ± 50	**40 ± 11.5**	**30 ± 6.2**
Radiatum	363 ± 33	323 ± 36	**45 ± 11.5**	**42 ± 9.0**
CA3				
Oriens	317 ± 26	303 ± 38	**26 ± 8.0**	**48 ± 6.6**
Pyramidal	477 ± 36	453 ± 42	**11 ± 5.6**	**32 ± 4.2***
Radiatum	328 ± 21	281 ± 32	**28 ± 9.5**	**40 ± 13.0**
Dentate gyrus				
Granular	467 ± 29	452 ± 50	**24 ± 6.1**	**48 ± 9.7***
Molecular	315 ± 49	278 ± 45	**34 ± 14.7**	**34 ± 9.5**
Mesencephalon				
Periaqueductal gray	496 ± 44	469 ± 45	**51 ± 15.0**	**47 ± 8.5**
Substantia nigra	456 ± 52	399 ± 38	**31 ± 10.5**	**37 ± 5.4**
**Basal ganglia/cholinergic forebrain**				
Globus pallidus	335 ± 43	347 ± 36	**46 ± 13.2**	**49 ± 12.5**
Striatum	339 ± 26	345 ± 21	**74 ± 15.4**	**62 ± 11.1**
NBM	535 ± 49	398 ± 31	**43 ± 9.2**	**11 ± 7.5***
Horiz. diagonal band	310 ± 25	327 ± 31	**121 ± 20.7**	**115 ± 17.6**
Vertical diagonal band	330 ± 25	399 ± 42	**135 ± 24.1**	**113 ± 23.2**
Medial septum	267 ± 24	284 ± 32	**152 ± 24.4**	**139 ± 23.7**

Data are mean ± S.E. M values of aCSF (n = 9) and SAP (n = 11) treated rats.
***** $p < 0.05$, when compared to aCSF group.

Table S3. [^{35}S]GTPγS basal and carbachol-induced (100 µM) binding in the different amygdaloid nuclei and several brain regions of vehicle (aCSF) and 192IgG-saporin-treated rats.

	Basal binding (nCi/g t.e.)		**Carbachol stimulation (% Over basal)**	
**Brain region**	aCSF	SAP	**aCSF**	**SAP**
Telencephalon				
**Amygdaloid nuclei**				
Anterior	421 ± 33	402 ± 29	**59 ± 9.1**	**47 ± 13.6**
Basolateral	487 ± 47	390 ± 44	**43 ± 6.6**	**48 ± 10.9**
Central	710 ± 91	525 ± 55	**27 ± 9.2**	**52 ± 7.8**
Lateral	483 ± 50	425 ± 43	**41 ± 9.3**	**36 ± 7.2**
Medial	720 ± 94	621 ± 69	**39 ± 8.5**	**34 ± 4.7**
Ventral subiculum	345 ± 29	294 ± 18	**37 ± 4.8**	**47 ± 6.7**
Rhinencephalon				
Lat olfactory tract	379 ± 45	324 ± 37	**82 ± 13.1**	**47 ± 11.5**
Midbrain				
AV thalamic nucleus	425 ± 32	397 ± 41	**232 ± 17.4**	**251 ± 28.6**
Gray sup colliculus	341 ± 27	362 ± 36	**197 ± 15.4**	**211 ± 19.9**
Rhomboencephalon				
Dorsal raphe	703 ± 103	590 ± 74	**19 ± 4.9**	**24 ± 4.7**
Locus coeruleus	220 ± 36	132 ± 16	**26 ± 11.9**	**78 ± 11.9**

Data are mean ± S.E. M values of aCSF (n = 9) and SAP (n = 11) treated rats.

Table S4. [^{35}S]GTPγS basal and carbachol-induced (100 μM) binding in different regions of grey matter from SHAM-operated rats.

	Basal binding (nCi/g t.e.)	**Carbachol stimulation (% Over basal)**		Basal binding (nCi/g t.e.)	**Carbachol stimulation (% Over basal)**
**Brain region**			**Brain region**		
Telencephalon			**Cerebral cortex**		
**Amygdala**			Cingulate	354 ± 30	**43 ± 12.7**
Anterior	403 ± 33	**37 ± 14.4**	Ectorhinal	337 ± 56	**36 ± 6.5**
Basolateral	387 ± 41	**53 ± 10.5**	Entorhinal	308 ± 30	**47 ± 7.1**
Central	536 ± 53	**32 ± 8.39**	Perirhinal	309 ± 43	**36 ± 13.5**
Lateral	373 ± 53	**78 ± 16.9**	Piriform	270 ± 31	**61 ± 9.8**
Medial	672 ± 84	**34 ± 11.7**	Somatosensory	368 ± 28	**34 ± 4.9**
**Hippocampus**			Motor	353 ± 37	**34 ± 14.8**
CA1			**Basal ganglia**		
Oriens	252 ± 25	**27 ± 12.0**	Globus pallidus	275 ± 25	**54 ± 12.2**
Pyramidal	366 ± 44	**55 ± 15.6**	Striatum	330 ± 37	**85 ± 14.0**
Radiatum	279 ± 19	**42 ± 9.8**	*Diencephalon*		
CA3			NBM	492 ± 51	**47 ± 12.3**
Oriens	280 ± 44	**21 ± 6.8**	Horiz diag band	356 ± 29	**115 ± 19.5**
Pyramidal	419 ± 52	**9 ± 6.2**	Vertical diag band	366 ± 28	**112 ± 25.6**
Radiatum	264 ± 38	**40 ± 19.8**	Medial septum	280 ± 50	**150 ± 41.2**
Dentate gyrus			*Rhinencephalon*		
Granular	437 ± 32	**25 ± 1.2**	Lat olfactory tract	356 ± 27	**41 ± 9.8**
Molecular	263 ± 45	**44 ± 9.2**	*Rhomboencephalon*		
Polimorphic	290 ± 39	**28 ± 15.7**	Dorsal raphe	533 ± 99	**42 ± 9.4**
Vent subic	301 ± 35	**49 ± 11.4**	Locus coeruleus	197 ± 30	**52 ± 6.0**
Brainstem			*Mesencephalon*		
Spinal trig N	178 ± 25	**87 ± 19.8**	Periaqueduc gray	450 ± 82	**47 ± 10.3**
			Substantia nigra	361 ± 56	**32 ± 14.9**

Data are mean ± S.E. M values of SHAM-operated (n = 6) rats.

Table S5. [^{3}H]-pirenzepine and [^{3}H]-oxotremorine binding in different brain regions of vehicle (aCSF) and 192IgG-saporin-treated rats.

[^{3}H]-pirenzepine binding (fmol/mg t.e.)			[^{3}H]-oxotremorine binding (fmol/mg t.e.)		
**Brain region**	aCSF	**SAP**	**Brain region**	aCSF	**SAP**
**Amygdala**			**Amygdala**		
Anterior	7.3 ± 0.8	3.6 ± 0.9*	Anterior	21.2 ± 2.7	31.1 ± 1.9
Basolateral	70.2 ± 5.9	74.5 ± 4.8	Basolateral	5.2 ± 1.7	5.1 ± 1.8
Central	30.5 ± 3.6	37.6 ± 6.6	Lateral	5.4 ± 1.1	5.3 ± 1.6
Lateral	66.1 ± 6.2	66.1 ± 4.8	**Hippocampus**		
**Hippocampus**			CA1		
CA1	111.3 ± 10	131.8 ± 8.8	Pyramidal	6.2 ± 1.5	7.5 ± 2.2
Oriens	114.8 ± 10	126.1 ± 4.2	CA3		
Pyramidal	127.9 ± 9.7	134.9 ± 7.9	Pyramidal	11.0 ± 2.3	11.3 ± 2.6
Radiatum	120.6 ± 8.6	136.2 ± 7.7	Dentate gyrus		
CA3	58.2 ± 6.6	64.1 ± 2.5	Granular	3.6 ± 1.5	7.1 ± 1.7
Oriens	43.4 ± 3.4	58.3 ± 2.3**	**Midbrain**		
Pyramidal	43.4 ± 4.5	53.5 ± 4.1	AV thalamic nucleus	66.3 ± 4.4	62.1 ± 2.7
Radiatum	49.5 ± 3.6	58.2 ± 3.6	Gray sup colliculus	57.8 ± 2.9	72.9 ± 4.5*
Dentate gyrus	108.1 ± 4.5	129.3 ± 5.0*	Opt sup colliculus	28.8 ± 5.3	31.3 ± 6.2
Granular	34.4 ± 2.5	46.4 ± 2.0*	**Cerebral cortex**		
Molecular	106.3 ± 8.8	114.9 ± 7.1	Cingulate	11.2 ± 2.3	17.9 ± 1.6*
Polimorphic	74.9 ± 5.5	79.7 ± 4.2	Ectorhinal	10.0 ± 2.1	14.6 ± 1.4
**Cerebral cortex**			Perirhinal	6.9 ± 2.3	10.7 ± 1.2
Cingulate	38.2 ± 1.0	36.1 ± 2.1	Motor		
Ectorhinal	58.5 ± 5.5	54.6 ± 2.9	Layer I-II	19.6 ± 2.6	26.5 ± 2.1
Entorhinal	51.4 ± 4.6	47.6 ± 3.7	Layer V-VI	12.7 ± 3.0	21.8 ± 2.4*
Perirhinal	55.2 ± 4.7	54.6 ± 3.0	Somatosensory		
Motor	39.5 ± 3.1	36.9 ± 1.3	Layer I-II	22.9 ± 1.1	29.7 ± 2.5
Somatosensory	43.7 ± 2.6	43.9 ± 2.6	Layer V-VI	12.2 ± 2.0	20.3 ± 2.5*
**Basal ganglia/cholinergic forebrain**			**Basal ganglia/cholinergic forebrain**		
Striatum	62.1 ± 6.1	63.8 ± 3.2	Striatum	8.2 ± 1.9	10.0 ± 1.5
Globus pallidus	5.0 ± 0.7	3.3 ± 0.5	Medial septum	30.1 ± 1.7	31.2 ± 3.6
NBM	5.4 ± 1.4	4.5 ± 0.7	NBM	7.2 ± 0.6	3.1 ± 0.4***

Data are mean ± S.E. M values of aCSF (n = 7) and SAP (n = 9) treated rats.
* $p < 0.05$, ** $p < 0.01$, *** $p < 0.001$ when compared to aCSF group.

Table S6. Absolute intensity of lipid species in organotypic cultures after treatments in both positive and negative ion mode.

			Intensity (u.a.)			
**Assignment**	**Cal *m/z***	**Exp *m/z***	**VEHICLE**	**CARBACHOL**	**CAR+SCOP**	**CAR+PIR**
**Positive ion mode**						
LPC (16:0) + H^+	496.3398	496.3379	$1.71 \cdot 10^9$ ± 224629	**$4.7 \cdot 10^9$ ± $6 \cdot 10^5$*a**	$3.1 \cdot 10^9$ ± $1 \cdot 10^5$	**4.25E+09 ± $6 \cdot 10^5$*b**
LPC (16:1) + H^+	494.3241	494.3270	3809. ± 461	**11696.00 ± 2506*a**	5400.00 ± 1336	**7250.00 ± 2101**
LPC (18:0) + H^+	524.3744	524.3711	910345 ± 166700	**$5.52 \cdot 10^9$ ± $5 \cdot 10^5$*a**	4. $\cdot 10^9$ ± $2 \cdot 10^5$	**$5.2 \cdot 10^9$ ± $6 \cdot 10^5$*b**
LPC (O-18:0) + H^+	508.3761	508.3788	6580 ± 864	**18654 ± 2098**a**	10133 ± 840	**15956 ± 1373*b**
LPC (O-18:1) + H+	506.3594	506.3605	$8.84 \cdot 10^4$ ± 13851	**$1.39 \cdot 10^9$ ± $2 \cdot 10^5$*a**	$9.5 \cdot 10^5$ ± $1 \cdot 10^5$	**$1.4 \cdot 10^9$ ± $1 \cdot 10^5$*b**
PC (36:4) + H^+	782.5694	782.5727	$9.29 \cdot 10^9$ ± $1 \cdot 10^9$	**$5.89 \cdot 10^9$ ± $3 \cdot 10^5$*a**	$7.1 \cdot 10^9$ ± $2 \cdot 10^5$	**$5.8 \cdot 10^9$ ± $2 \cdot 10^5$**b**
PC (O-36:4) + H^+	768.5902	768.5943	173714 ± 7706	**54424 ± 9721*a**	146795 ± 6801	**69580 ± 12002*b**
PC (36:5) + Na^+	802.5357	802.5401	44361 ± 1268	**23056 ± 3204**	35767 ± 6284	**14701 ± 3088**b**
PC (38:5) + H^+	808.5851	808.5861	761567 ± 42629	**526654 ± 21651*a**	700222 ± 63349	**568492 ± 48686**
PC (38:6) + H^+	806.5694	806.5742	764787 ± 81976	**349308 ± 23788**a**	481258 ± 32195	**349138 ± 4729*b**
PC(38:7) + H^+	804.5565	804.5538	597052 ± 100617	**270280 ± 26512*a**	402153 ± 59147	**248105 ± 40459*b**
PC(O-38:7) + H^+	790.5745	790.5795	63971 ± 5613	**26141 ± 4941*a**	64351 ± 9306	**30403 ± 4465**
PC(40:7) + Na^+/PC (36:4) + H^+	832.5851	832.5851	288228 ± 24796*	**197092 ± 19490**	282757 ± 21872	**185810 ± 35558**
**Negative ion mode**						
LPC (O-18:0) -CH_3	508.3409	508.3418	3997 ± 1106	**18226 ± 1697*a**	11564 ± 1505	**20301 ± 3041**b**

The maximal peak in negative ion mode, PI (20:4/18:0), changed with treatments. Data are mean ± S.E.M values of absolute intensity of VEHICLE (n = 6), CARBACHOL (n = 4), CAR+SCOP: Carbachol+Scopolamine (n = 4), and CAR+PIR: Carbachol+Pirenzepine (n = 4) of absolute intensity. *$p < 0.05$, **$p < 0.01$. **a*** when compared VEHICLE *vs* CARBACHOL. **b*** when compared VEHICLE *vs* CAR+PYR. PC: phosphatidylcholine; LPC: phosphatidylcholine; Cal: calculated; Exp: experimental.
